# Substrate‐Inspired Fragment Merging and Growing Affords Efficacious LasB Inhibitors

**DOI:** 10.1002/anie.202112295

**Published:** 2021-12-13

**Authors:** Cansu Kaya, Isabell Walter, Samir Yahiaoui, Asfandyar Sikandar, Alaa Alhayek, Jelena Konstantinović, Andreas M. Kany, Jörg Haupenthal, Jesko Köhnke, Rolf W. Hartmann, Anna K. H. Hirsch

**Affiliations:** ^1^ Helmholtz Institute for Pharmaceutical Research Saarland (HIPS) Helmholtz Centre for Infection Research (HZI) Campus E8.1 66123 Saarbrücken Germany; ^2^ Department of Pharmacy Saarland University Campus E8.1 66123 Saarbrücken Germany; ^3^ Helmholtz Institute for Pharmaceutical Research Saarland (HIPS) Helmholtz Centre for Infection Research (HZI) Campus E8.1 66123 Saarbrücken Germany

**Keywords:** Antibiotic resistance, Antivirulence, Fragment merging, Mercaptoacetamides, *Pseudomonas aeruginosa*

## Abstract

Extracellular virulence factors have emerged as attractive targets in the current antimicrobial resistance crisis. The Gram‐negative pathogen Pseudomonas aeruginosa secretes the virulence factor elastase B (LasB), which plays an important role in the infection process. Here, we report a sub‐micromolar, non‐peptidic, fragment‐like inhibitor of LasB discovered by careful visual inspection of structural data. Inspired by the natural LasB substrate, the original fragment was successfully merged and grown. The optimized inhibitor is accessible via simple chemistry and retained selectivity with a substantial improvement in activity, which can be rationalized by the crystal structure of LasB in complex with the inhibitor. We also demonstrate an improved in vivo efficacy of the optimized hit in Galleria mellonella larvae, highlighting the significance of this class of compounds as promising drug candidates.

## Graphical abstract

An efficient fragment merging and growing strategy with simple chemistry yielded a fragment‐like inhibitor of the virulence factor LasB from *Pseudomonas aeruginosa* with a twelve‐fold boost in activity. Our optimized structure shows an improved in vivo efficacy demonstrating the significance of exploiting alternative binding modes to succeed in simple fragment merging. The graphic was created with the software tool BioRender (www.biorender.com).

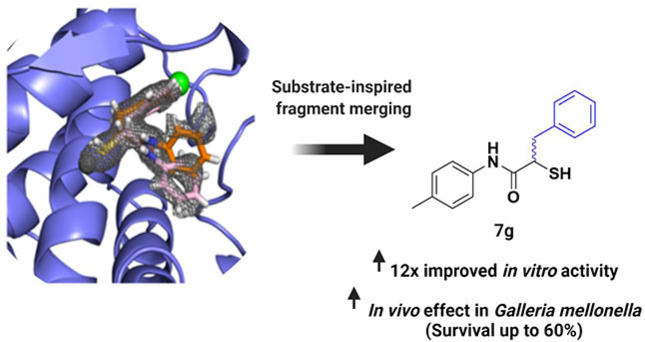

Alternative binding modes are often observed in the realm of fragment‐based drug design.^[1]^ Despite the potential to significantly accelerate hit‐to‐lead optimization, there are few examples of successful fragment linking/merging or systematic exploitation of such invaluable sources of structural information. This is presumably due to a number of conditions that need to be met such as the linker composition and the resulting ADMET properties.[^2^, ^3^] We propose that a careful focus on the the structural data of fragments could serve as an important starting point to facilitate optimization via linking/merging, ensuring favorable properties. We explore this hypothesis with simple chemistry using a virulence factor from *Pseudomonas aeruginosa*.

*P. aeruginosa* is a Gram‐negative bacterium that is ranked amongst the most critical pathogens by the World Health Organization.^[4]^ This opportunistic bacterium causes ≈10% of hospital‐acquired infections and has a high incidence in immunocompromised and cystic‐fibrosis patients.[^5^, ^6^, ^7^, ^8^] It has an especially low permeability of the outer membrane, which prevents the entry of antibiotics into the cell.^[9]^ In addition, its efflux pumps efficiently move undesired antimicrobials out of the cell and its β‐lactamases are able to inactivate the corresponding β‐lactam antibiotics[^10^, ^11^, ^12^, ^13^] contributing to the emergence of drug‐resistant *P. aeruginosa* strains.[^14^, ^15^]

In the search for anti‐infectives with novel modes of action, addressing bacterial virulence has become a widely applicable method.[^16^, ^17^, ^18^] Virulence factors are used by pathogenic bacteria and act through several mechanisms, including the invasion of host cells, biofilm formation and the evasion of the host immune response.^[19]^ Inhibition of virulence factors reduces bacterial virulence and enables clearance of the pathogens by either the host immune system or antibiotics.[^20^, ^21^] The main advantages of this strategy is the reduced selective pressure on the bacteria, which decreases the risk of resistance development, and the fact that commensal bacteria remain unaffected.^[20]^ Among other virulence factors, the secreted metalloprotease LasB has been validated as one of the most important components contributing to the virulence of *P. aeruginosa*.^[22]^ LasB is thus a particularly attractive target and addressing it circumvents permeation and efflux issues due to its extracellular location.

LasB plays a crucial role in the pathogenic invasion of tissues and is predominantly responsible for acute nosocomial infections.[^19^, ^23^] It has the ability to degrade elastin, an important component of lung tissue, blood vessels and skin, which makes it a key target for inhibition. Until now, natural products such as the streptomyces metalloprotease inhibitor (SMPI) from *Streptomyces nigrescens* TK‐23^[24]^ and phosphoramidon (Pam)^[25]^ (Figure 1, compound **1**), small peptides containing metal‐chelating motifs such as thiol,[^26^, ^27^, ^28^] hydroxamate^[29]^ or carboxylic acid[^30^, ^31^] groups (Figure 1, compound **2**), have been reported. However, most of these inhibitors show poor selectivity with respect to mammalian metalloenzymes. The small synthetic molecules with hydroxamate and mercaptoacetamide groups that have been reported by us (Figure 1, compounds **3** and **4**) are promising LasB inhibitors with better selectivity profiles, yet there are still substantial improvements necessary on the way to clinically applicable drugs.[^32^, ^33^]

**Figure 1:**
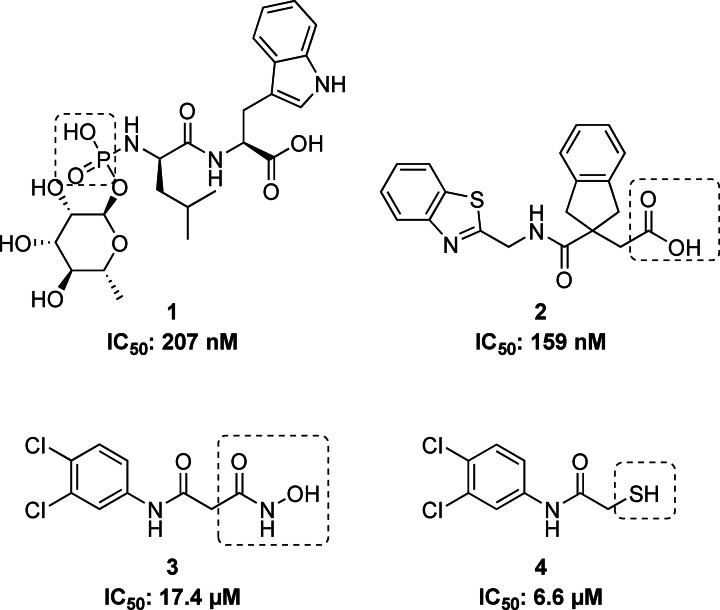
Structures of known LasB inhibitors. Zinc‐binding residues are highlighted.

We set out to exploit alternative binding modes of the parent hit to guide efficient fragment merging and growing and to overcome the above‐mentioned limitations of the reported inhibitors. We present the structure‐based optimization of a fragment‐like LasB inhibitor that resulted in a twelve‐fold boost in activity combined with improved in vivo efficacy. The inhibitory potency was determined in vitro whilst ensuring the derivatives displayed no direct antimicrobial activity. Having confirmed the exquisite selectivity using a panel of representative human off‐targets, we analyzed the efficacy in vivo in *Galleria mellonella* larvae. A LasB‐inhibitor complex crystal structure verified the predicted binding mode.

We recently reported the crystal structure of the hit compound **4** (IC_50_=6.6±0.3 μM) in complex with LasB.^[33]^ To our surprise, two molecules of **4** were present in the substrate binding pocket of LasB, which set the stage for rational compound optimization by merging both molecules of **4**. Our attempts to combine these two molecules into one *N*‐benzylamide derivative did not provide the desired activity.^[33]^ In our next attempt to optimize the molecular interactions in the binding pocket, we shifted the benzyl moiety to the alpha position of the amide moiety to create a non‐peptidic substrate mimick (Phe side‐chain), which afforded the first derivative of the α‐alkyl‐*N*‐aryl mercaptoacetamide class. Compound **7 a** displayed a two‐fold increase in activity (Table 1).

**Table 1:** In vitro activities of α‐alkyl‐*N*‐aryl mercaptoacetamides and α‐benzyl‐*N*‐aryl mercaptoacetamides against LasB.^[a]^ 
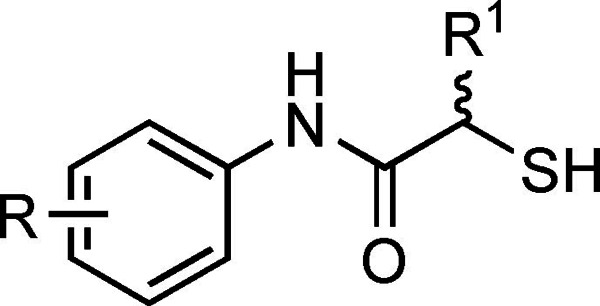

Compound	R	R^1^	IC_50_ [μM]
**4**	3,4‐di‐Cl	H	6.6±0.3
**7 a**	3,4‐di‐Cl	Benzyl	2.7±0.4
**7 b**	3,4‐di‐Cl	Cyclohexylmethyl	12±3
**7 c**	3,4‐di‐Cl	Cyclopropylmethyl	6.3±1.2
**7 d**	H	Benzyl	1.2±0.1
**7 e**	2‐Me	Benzyl	2.4±1.0
**7 f**	3‐Me	Benzyl	1.0±0.4
**7 g**	4‐Me	Benzyl	0.48±0.04

[a] Means±standard deviations of at least two independent experiments.

To explore to which degree the benzyl moiety is responsible for the increase in activity, we further investigated the effect of cyclohexylmethyl and cyclopropylmethyl side chains, while maintaining the di‐chloro substituent on the *N*‐aryl ring. Although the IC_50_ value of compound **7 c** remained similar to compound **4** with a relatively small‐sized cyclopropylmethyl group, it increased significantly for compound **7 b** with a cyclohexylmethyl substituent (Table 1). Therefore, we concluded that both optimal filling of the unoccupied space and the aromaticity of the benzyl group contributed to the activity.

Our next step was to investigate the importance of the di‐chloro motif that is engaged in key hydrophobic interactions in the binding pocket. Compared to **7 a**, compound **7 d** showed a further two‐fold improvement in IC_50_ value, which demonstrated that the presence of a di‐chloro substituent was not essential for the activity of the α‐benzyl‐*N*‐aryl mercaptoacetamide class.

To elucidate the binding mode of α‐substituted mercaptoacetamides, we co‐crystallized compound **7 d** with LasB (Figure 2A). Full details of the data collection and refinement statistics can be found in the Supporting Information (Table S1). As expected, the compound was found in the same binding pocket as previously reported for *N*‐(3,4‐dichlorophenyl) mercaptoacetamide hit **4** and occupied the S1′–S2′ pockets with the thiol group coordinating the active‐site Zn^2+^ cation (Figure 2B, Figure S1). The *N*‐arylacetamide group is stabilized by H‐bonding and hydrophobic interactions (Figure S2). The ligand is possibly anchored in the binding pocket by the carbonyl oxygen, which forms a bidendate hydrogen bond with Arg198. The phenyl group of the *N*‐arylacetamide occupies the wide, open and solvent‐accessible entrance of the S1′ binding pocket, which may explain the two different orientations observed for this part of the compound in the crystal structure (Figure 2). The benzyl group lies in the lipophilic S2′ binding pocket and is stabilized by numerous hydrophobic interactions (Figure S2).

**Figure 2:**
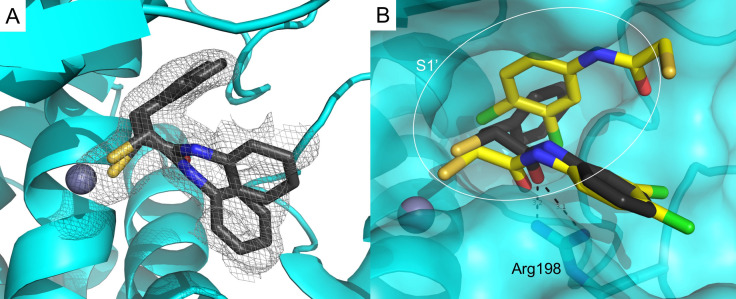
A) Crystal structure of LasB in complex with **7 d** (PDB code: 7OC7). Cartoon representation of LasB (cyan) in complex with **7 d** (black). The gray isomesh represents the polder map of **7 d** contoured at 2 σ. Two different states of **7 d** with different occupancies are observed. B) Superposition of LasB (cyan surface) structures in complex with **4** (yellow) or **7 d** (black, major conformation shown) showing the phenyl group occupying the S1′ binding‐site of the enzyme. The active‐site Zn^2+^ cation is shown as a gray sphere.

In contrast to the binding mode of compound **4**, only one molecule of compound **7 d** binds to the protein, which leads to the closure of the binding pocket – a phenomenon generally observed for thermolysin‐like proteases like LasB upon inhibitor binding.^[34]^ Our structure‐based strategy to move the benzyl moiety from the amide nitrogen to the alpha position proved to be successful, as we were able to occupy the space in the binding pocket with one molecule.^[33]^

The LasB–**7 d** crystal structure provided a deeper understanding of the potential interactions in the surrounding unoccupied space and paved the way for further optimization. For example, the tolerance of other lipophilic substituent(s), especially in the S1′ pocket, is well‐documented. As we previously discovered that the hydrophobic di‐chloro motif is not essential for the improvement in activity, our next choice for a lipophilic substituent was a sterically less demanding methyl group. We used the crystal structure for a focused, structure‐based optimization study. As supported by the docking pose (Figure S3), the presence of a lipophilic methyl substituent in the *para* position most likely leads to further strengthening of the hydrophobic interactions with Leu197, and in combination with the benzyl group in the alpha position, provides optimal interactions in the binding pocket. Taking this into account, we synthesized three regioisomers and all of them proved to be more potent than the initially optimized structure, compound **7 a** (Table 1). Expectedly, the introduction of a *para* methyl substituent on the aromatic core of the *N*‐arylacetamide group had the most profound effect on activity, confirming the beneficial inhibitor–protein interactions. These interactions account for the submicromolar activity observed for compound **7 g** (IC_50_=0.48±0.04 μM).

The synthetic route for all derivatives is shown in Scheme 1. Diazotization and subsequent chlorination of the corresponding commercially available racemic amino acids **4 a**–**4 c** yielded α‐chloro carboxylic acids. Their coupling with the respective aniline gave the intermediates **5 a**–**5 g**. The thioacetate function was introduced via an S_N_2 reaction using potassium thioacetate **6 a**–**6 g**. Deprotection of the thioacetate under basic conditions afforded final compounds **7 a**–**7 g** in 20–88% yield as free thiols.

**Scheme 1:**
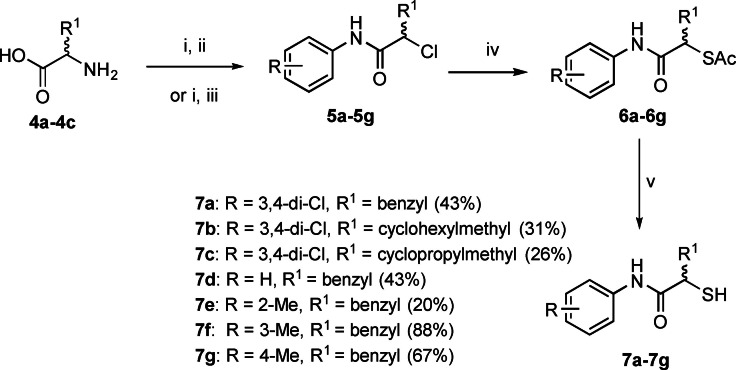
Synthetic scheme of α‐alkyl/aryl compound class.^[a]^

Prior to investigating the in vivo effect of our improved inhibitors, we analyzed their antibacterial activity. A minimum inhibitory concentration (MIC) assay revealed no direct effect on pathogen growth by inhibitor **7 d** (Supporting Information, Page S3, MIC>100 μM). Furthermore, we explored its cytotoxicity towards three human cell lines (Supporting Information, Page S3, IC_50_>100 μM). Given that poor selectivity towards metalloproteases is often an issue, we investigated selectivity of selected inhibitors for LasB over six matrix metalloproteases (MMPs) and additional three human off‐targets (Table S2). While the selectivity of the optimized compounds **7 d** and **7 g** could be maintained compared to the hit compound **4** with the exception of TACE, a slightly reduced cytotoxicity of the novel compounds was shown.

We next analyzed the antivirulence activity of LasB inhibitors in vivo, using a simple model based on *G. mellonella* larvae. This method is used to evaluate the treatment options for *P. aeruginosa*‐induced infections and to demonstrate the efficacy of LasB inhibitors in preparation for a murine in vivo pharmacodynamics study.[^32^, ^33^] We injected the larvae with the supernatant (s.n.) of *P. aeruginosa* PA14 with or without 0.25 nmol of either **4** or **7 g**, incubated them for three days and recorded the survival once per day (Figure 3). Our results showed that PA14 s.n. reduced the survival of larvae by up to 88% after three days of incubation. Compound **4**, which was used as a control, showed virtually no improvement in survival after three days. Treatment of larvae with 0.25 nmol of compound **7 g** increased survival up to 60% compared to PBS injected larvae. These results validate our inhibitors as promising candidates to block the pathogenicity of *P. aeruginosa* and confirm that the boost in inhibitory activity in vitro translates to an improved in vivo effect.

**Figure 3:**
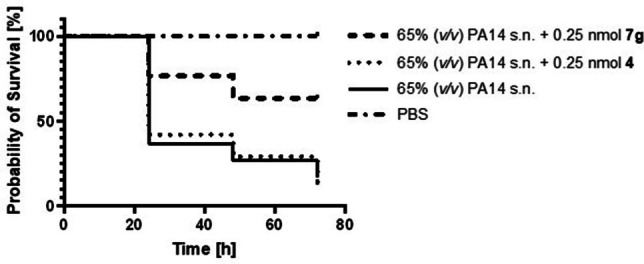
Kaplan–Meier survival analysis of larvae treated with 0.25 nmol compound **4** (dotted) (*p*≤0.9452), 0.25 nmol compound **7 g** (two dash) (*p*≤0.0002) or PA14 s.n. only (solid) (*n*=3). PA14 s.n. was also present during compound treatment. PBS served as a negative control (dot dash). Compounds **7 g** and **4** in PBS showed 100% survival. s.n.: supernatant.

In summary, by utilizing structure‐guided fragment merging/linking inspired by the natural substrate, we achieved a substantial increase in potency of our LasB inhibitors that translated into in vivo efficacy. We identified compound **7 g**, which showed a twelve‐fold improvement in activity compared to our best previously reported inhibitor. Encouraged by the excellent in vitro activity of these compounds, we also demonstrated an in vivo effect in a *G. mellonella* model. The survival rate of larvae infected with PA14 supernatant and treated with compound **7 g** were significantly improved as compared to our previous inhibitor **4**.

Although structure‐guided exploration of the *N*‐aryl ring substitution provided us with a strong starting point for lead optimization, systematic variation of the substituent in the α‐position (R^1^) should be considered to exploit the lipophilicity of the S2′ pocket. In addition, compound **7 g** only occupies a small fraction (approx. 24%) of the total predicted binding pocket volume, leaving the S2′ pocket largely untouched (Figure S4). Therefore, extension of the thiol group in that direction should allow additional ligand‐protein interactions, leading to a further improved inhibitory potency. Calculation of Ligand Efficiency (LE) and Lipophilic Ligand Efficiency (LLE) for compound **4** (LE:0.44, LLE:1.67) and our optimized hit **7 g** (LE:0.43, LLE:2.37) revealed a slight improvement in LLE with no change in LE, thus representing a successful starting point for further optimization and tuning of this class.

Our work demonstrates the significance of exploiting alternative binding modes to succeed in simple fragment merging. Substrate‐inspired design led to an improved potency of previously identified structures accompanied by an enhanced efficacy in vivo, thereby accelerating the translational path. This concept should be applicable to other targets and lays an important foundation for the future development of this class of inhibitors, as they hold the potential to become promising candidates for therapeutic use and to deliver the proof‐of‐concept of small synthetic molecules targeting virulence factors in the clinic.

## Supporting information

As a service to our authors and readers, this journal provides supporting information supplied by the authors. Such materials are peer reviewed and may be re‐organized for online delivery, but are not copy‐edited or typeset. Technical support issues arising from supporting information (other than missing files) should be addressed to the authors.

Supporting Information
