# Supplementary material for: Substrate‐Inspired Fragment Merging and Growing Affords Efficacious LasB Inhibitors
Source: Angew Chem Int Ed Engl. 2021 Dec 13;61(5):e202112295. doi: 10.1002/anie.202112295 (PMC9299988; doi:10.1002/anie.202112295)
Supplement: Supplementary file 1 — Supporting Information [file ANIE-61-0-s001.pdf]

Angewandte Chemie
Eine Zeitschrift der Gesellschaft Deutscher Chemiker

Supporting Information

Substrate-Inspired Fragment Merging and Growing Affords Efficacious LasB Inhibitors

*C. Kaya, I. Walter, S. Yahiaoui, A. Sikandar, A. Alhayek, J. Konstantinović, A. M. Kany, J. Haupenthal, J. Köhnke, R. W. Hartmann, A. K. H. Hirsch**

Table of Contents

General Information **S2**

Figures and Tables **S5**

Synthesis of intermediate and final compounds **S12**

NMR and LC-MS spectra of final compounds **S21**

References **S46**

General Information

LasB Inhibition Assay. The purification of LasB from *P. aeruginosa* P14 supernatant and the subsequent performance of the FRET-based *in vitro* inhibition assay were performed as described previously.[1]

Antibacterial Activity. Minimum inhibitory concentration (MIC) assays were performed in *P. aeruginosa* PA14 as described previously.[1] The MIC value was higher than 100 μM for compounds **4**, **7d** and **7g**. For all compounds, at 100 µM, the bacterial growth was reduced by less than 10%.

Inhibition Assays with human off-targets. Assays focusing on the inhibition of human MMPs and ADAM17 were performed as described previously.[2,3]

Cytotoxicity Assay. The toxicity of selected compounds towards three cell lines was determined as described previously. Compounds **4**, **7d** and **7g** showed no relevant cytotoxic behaviour against the human hepatoma cell line (HepG2), human embryonic kidney (HEK) 293 cells and adenocarcinomic human alveolar basal epithelial cells (A549) with IC_{50} values higher than 100 μM.[1,4] In detail, while compound **4** showed a very low reduction in the viability of HepG2 (23±3%) and HEK cells (33±10%) at 100 µM, all other inhibition values were found to be below 10%.

X-Ray Crystallography. LasB was expressed and purified as described previously.[1] The protein was concentrated to 12 mg/mL and mixed with compound **7d** at a final concentration of 1 mM. Complex crystals were obtained in 0.1 M sodium acetate pH 4.6, and 15% (*w/v*) PEG 20,000. Crystals were cryoprotected in glycerol, and diffraction data was collected from single crystals at 100 K at beamline ID30A-3 (ESRF) at a wavelength of 0.967 Å. Data were processed using Xia2 or XDS, and the structure solved using PHASER Molecular Replacement with *P. aeruginosa* elastase (PDB ID 1EZM) as a search model.[5–7] The models were manually rebuilt with COOT and refined using PHENIX and Refmac5.[8–10]

Molecular Modeling. Modeling was performed as described previously.[1]

Preparation of *P. aeruginosa* supernatant and LasB activity evaluation. A culture of a single colony of PA14 was grown in lysogeny broth medium at 28 °C with constant shaking at 130 rpm for 3 days. Then, the culture was centrifuged at 4 °C, 5000 rpm for 30 minutes. Finally, the supernatant was passed through a membrane filter of 0.2 μM to sterilize it. The supernatant

was aliquoted and stored at –80 °C until usage. The LasB activity of the supernatant was evaluated using the FRET-based assay described previously.[1]

***In vivo Galleria mellonella* virulence assay.** *G. mellonella* larvae were purchased from BioSystems Technology (Exeter, United Kingdom), stored at 4 °C in the dark and used within 2 weeks. Prior to injection, larvae were immobilized by incubation for 10–15 min on ice. Then, the injection was performed using a LA120 syringe pump (Landgraf Laborsysteme, Langenhagen, Germany) supplied with a 1 mL syringe (B. Braun, Melsungen, Germany) and Sterican 0.30 × 12 mm, 30G × 1.5 sterile needles (B. Braun). The larvae were injected with 10 μL of sample into the last right proleg. The larvae were classified into various groups based on the applied treatment. Two negative control groups supplemented with no injection to control the quality of larvae and a buffer control group injected with sterile PBS were included. A positive control group was also included, and the larvae were administered with 65% (*v/v*) PA14 supernatant. To test the anti-virulence effect of LasB inhibitors, a mixture of 65% (*v/v*) PA14 supernatant, LasB inhibitor and 300 μM TCEP were incubated at 37 °C for 30 min and injected into the larvae. All groups were incubated at 37 °C and inspected once per day for 4 days post-treatment and to record mortality. The larvae were considered dead if they are black and do not move when stimulated by contact with the forceps. The survival analysis was performed using GraphPad Prism v8, data were plotted using the Kaplan-Meier method, and statistical significance between groups was calculated with log-rank test.

General Chemistry. All reagents were used from commercial suppliers without further purification. Procedures were not optimized regarding yield. NMR spectra were recorded on a Bruker AV 500 (500 MHz) spectrometer at room temperature. Chemical shifts are given in parts per million (ppm) and referenced against the residual proton, ^{1}H, or carbon, ^{13}C, resonances of the >99% deuterated solvents as internal reference. Coupling constants (*J*) are given in Hertz (Hz). Data are reported as follows: chemical shift, multiplicity (s = singlet, d = doublet, t = triplet, dd = doublet of doublets, dt =doublet of triplets, m = multiplet, br = broad and combinations of these) coupling constants and integration. Liquid chromatography-mass spectrometry (LC-MS) was performed on an LC-MS system, consisting of a DionexUltiMate 3000 pump, autosampler, column compartment, and detector (Thermo Fisher Scientific, Dreieich, Germany) and ESI quadrupole MS (MSQ Plus or ISQ EC, Thermo Fisher Scientific, Dreieich, Germany). High-resolution mass spectra were determined by LC-MS/MS using Thermo Scientific Q Exactive Focus Orbitrap LC-MS/MS system. Purity of the final

compounds was determined by LC-MS using the area percentage method on the UV trace recorded at a wavelength of 254 nm and found to be >95%.

Figures and Tables

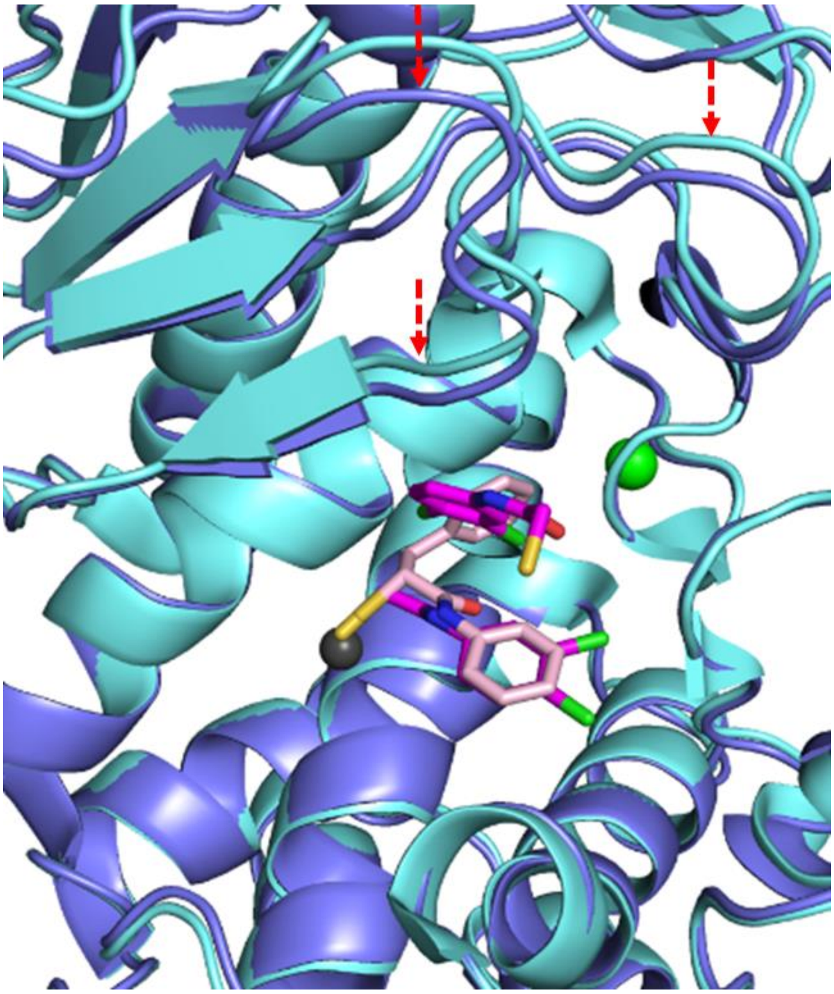

Figure S1. Comparison of compound **7g** and compound **4** binding to LasB. Superposition of the LasB-Compound **4** (aqua; PDB 6f8b) and LasB-**7g** (slate) structures are shown. The movement of loop leading to closure of the binding pocket upon binding to **7g** (light pink sticks) is highlighted by dotted arrows. For simplicity, only the major conformation of **7g** observed in the crystal structure in shown. The color scheme used: **4** (magenta), **7g** (light pink), Zn^{2+} (gray) and Ca^{+2} (green).

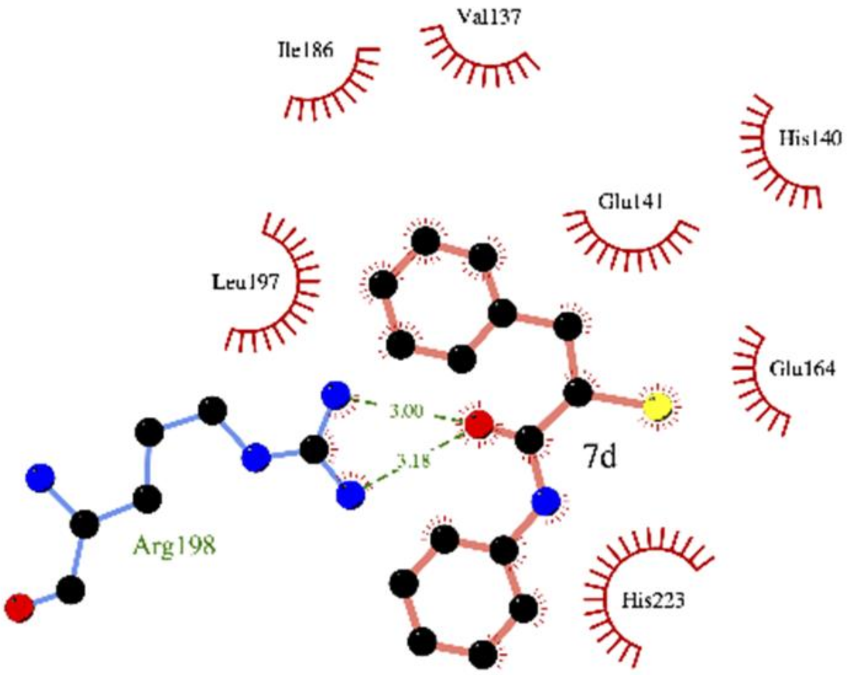

Figure S2. Schematic 2-D representation of LasB-**7d** complex created with LIGPLOT.

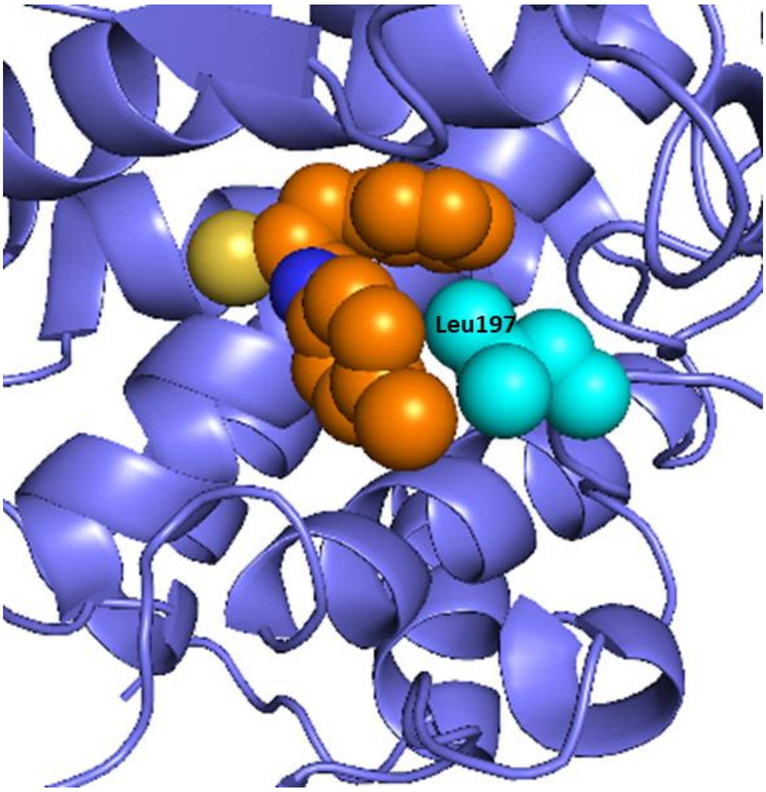

Figure S3. Modelling of *para*-methyl substituted derivative of compound **7d** in the LasB ligand binding pocket.

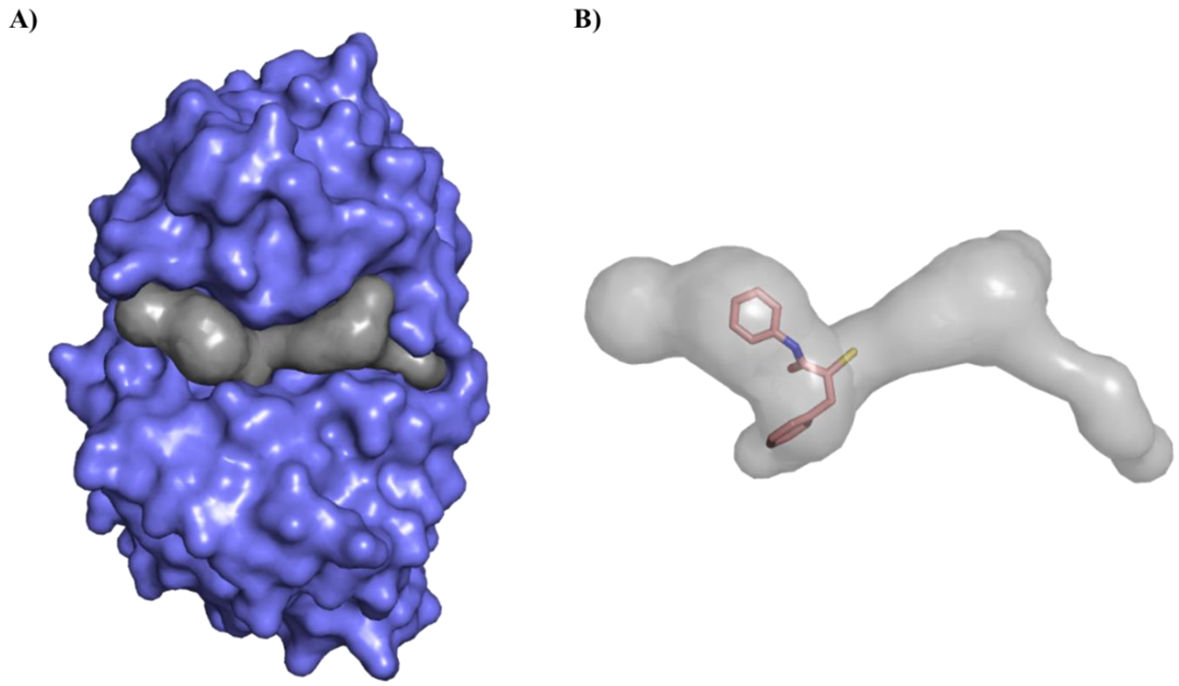

Figure S4. Analysis of putative tunnel identified by CAVER in the structure of LasB in complex with compound **7g. A)** LasB (slate) and the computed tunnel (grey) are shown as surface representation. **B)** Surface diagram showing the position of **7g** in the tunnel. Total volume of the tunnel and **7g** was calculated to be approximately 360 Å^3 and 86 Å^3 .

Table S1. Data collection and refinement statistics.

LasB_7d	
**PDB ID**	7OC7
**Data collection**	
Space group	P 1 2_1 1
Cell dimension	
a, *b*, *c* (Å)	39.4, 92.5, 40.76
α, β, γ (°)	90.0, 114.0, 90.0
Wavelength (Å)	0.96768
Resolution	1.95 (2.00 – 1.95) *
R_{sym} or R_{merge}	0.044 (0.141)
R_{pim}	0.029 (0.092)
CC (1/2)	0.99 (0.985)
I / σI	26.7 (10.2)
Completeness (%)	95.1 (94.1)
Redundancy	6.0 (6.1)
**Refinement**	
Resolution (Å)	37.21 – 1.95
No. reflection	18485
R_{work} / R_{free}	0.198 / 0.239
No. atoms	2,637
Protein	2,283
Ligands	38
Solvent	316
Protein residues	298
B-factors	17.15
Protein	16.10
Ligands	22.12
Water	24.16

R. m. s deviations	
Bond length (Å)	0.010
Bond angels (°)	0.84
MolProbity score	1.52

*Values in parentheses are for highest-resolution shell.

Table S2. Selectivity of selected inhibitors (n.i. = <10% inhibition).

	**4**	**7d**	**7g**
**MMP-1**	n.i.	n.i.	n.i.
**MMP-2**	n.i.	n.i.	n.i.
**MMP-3**	n.i.	n.i.	n.i.
**MMP-7**	n.i.	n.i.	n.i.
**MMP-8**	n.i.	26 ± 4	n.i.
**MMP-14**	n.i.	n.i.	n.i.
	**IC_{50} [μM]**		
**ADAM17**	>100	2.2 ± 0.1	4.8 ± 1.5
**HDAC-3**	>100	>100	>100
**HDAC-8**	>100	>100	>100

Synthesis of intermediate and final compounds

General procedure i: Synthesis of chloro acid derivatives 4a–4c from the corresponding amino acid

Amino acid (1.0 eq) was dissolved in 6 N HCl (2 mL/mmol or until mostly dissolved) under nitrogen atmosphere and cooled to -5 °C. $NaNO_2$ (1.5–2.5 eq) was dissolved in water (0.3 mL/mmol amino acid) and added dropwise slowly. The mixture was stirred overnight while warming to r.t. The reaction mixture was extracted with EtOAc/THF (3:1). The combined organic extracts were washed with saturated aq. NaCl solution and dried over anh. Na_2SO_4 and filtered. The solvent was removed under reduced pressure to afford the product. The crude was used in the next step without further purification.

General procedure ii: Synthesis of derivatives 5a, 5d–5g

The acid (1.0 eq), $SOCl_2$ (2.0 eq) and a few drops of DMF were heated to 70 °C for 1 h. The cooled mixture was added dropwise to a solution of the corresponding aniline (1.1 eq) in DMF (1 mL/mmol) a cooled to 0 °C. The mixture was stirred at r.t overnight. The reaction was quenched with water and extracted with EtOAc (3x). The combined organic extracts were washed with saturated aq. NaCl solution, dried over anh. Na_2SO_4 and filtered. The solvent was removed under reduced pressure to afford the crude product. The purification was done by column chromatography or flash chromatography.

General procedure iii: Synthesis of derivatives 5b and 5c

2-Chloro-3-cyclohexylpropanoic acid or 2-chloro-3-cyclopropylpropanoic acid (1.2 eq) and EDC·HCl (1.2 eq) were added to a solution of the corresponding aniline (1.0 eq) in DCM. The resultant mixture was stirred at r.t. for 3–4 h. The reaction was monitored with TLC or LC-MS. The solution was washed with 1 M HCl followed by saturated aqueous NaCl solution (1x) then dried over anh. Na_2SO_4. The organic phase was filtered and concentrated under reduced pressure to afford the crude product. The crude was used in the next step without further purification.

General procedure iv: Synthesis of thioacetate derivatives 6a–6g

The amide (1.0 eq) was dissolved in acetone under argon atmosphere. To this solution, CH_3COSK (1.5–2.0 eq) was added, and the reaction was stirred at r.t. for 2–6 h. It was monitored by TLC or LC-MS. The reaction was quenched with water and extracted with EtOAc

(3x). The combined organic extracts were washed with saturated aq. NaCl solution (1x), dried over anh. Na_2SO_4 and filtered. The solvent was removed under reduced pressure to afford the crude product. The purification was done by flash chromatography.

General procedure v: Hydrolysis of thioacetate for derivatives 7a–7g

The thioacetate (1.0 eq) was dissolved in methanol (5 mL/mmol) under argon atmosphere and 2 M aqueous NaOH solution (2.0 eq) or solid NaOH (3.0 eq) was added. The reaction was stirred at r.t. for 1–3 h before quenching with 1 M or 2 M HCl. The reaction was extracted with EtOAc and washed with 0.5 M HCl. The combined organic extracts were washed with saturated aqueous NaCl solution (1x) and then dried over anh. Na_2SO_4. The solvent was removed under reduced pressure to afford the crude product. The purification was done by column chromatography or preparative HPLC (H_2O+0.05%FA/ACN+0.05%FA 95:5 → 5:95).

2-Chloro-3-phenylpropanoic acid (4a)

Compound **4a** was prepared according to **general procedure i**, using DL-phenylalanine (1 g, 6.0 mmol) and $NaNO_2$ (1.46 g, 21.2 mmol). The crude product was obtained as light yellow oil and used without further purification (1.05 g, 94%). ^{1}H NMR (500 MHz, $CDCl_3$) δ ppm: 7.37–7.24 (m, 5H), 4.51 (dd, *J* = 7.8, 6.9 Hz, 1H), 3.42 (dd, *J* = 14.0, 6.7 Hz, 1H), 3.21 (dd, *J* = 14.1, 7.9 Hz, 1H). MS (ESI$^-$) *m/z* 183.25 [M–H]$^-$, 147.23 [M–H–HCl]$^-$.

2-Chloro-*N*-(3,4-dichlorophenyl)-3-phenylpropanamide (5a)

Compound **5a** was prepared according to **general procedure i**, using compound **4a** (350 mg, 1.90 mmol), $SOCl_2$ (275 µL, 3.8 mmol) and 3,4-dichloroaniline (339 mg, 2.1 mmol). Purification was done by flash chromatography (Hex/EtOAc, 100:0 to 0:100). The final product was obtained as white solid (388 mg, 62%). ^{1}H NMR (500 MHz, $CDCl_3$) δ ppm: 8.03 (s, 1H), 7.71 (d, *J* = 2.5 Hz, 1H), 7.39 (d, *J* = 8.7 Hz 1H), 7.36–7.23 (m, 6H), 4.68 (dd, *J* = 7.6, 4.5 Hz, 1H), 3.50 (dd, *J* = 14.3, 4.5 Hz, 1H), 3.31 (dd, *J* = 14.3, 7.6 Hz, 1H). ^{13}C NMR (126 MHz, $CDCl_3$) δ ppm: 166.5, 136.2, 135.7, 133.1, 130.7, 129.8, 128.7, 127.6, 122.0, 119.5, 61.8, 41.4. MS (ESI$^+$) *m/z* 330.09 [M+H]$^+$.

2-Chloro-*N*-(3’,4’-dichlorophenyl)-3-cyclohexylpropanamide (5b).

Compound **5b** was synthesized in two steps. The first step was performed according to **general procedure i**, using DL-3-cyclohexylalanine (260 mg, 1.52 mmol) and $NaNO_2$ (262 mg, 3.80 mmol). The obtained crude product **4b** was used without further purification. The second step

was achieved according to the **general procedure iii**, using the obtained crude product **4b** from the first step, 3,4-dichloroaniline (205 mg, 1.27 mmol), EDC·HCl (291 mg, 1.52 mmol) and CH_2Cl_2 (10 mL). The reaction was stirred overnight at room temperature. The crude product was purified using column chromatography (100% CH_2Cl_2). The product **5b** was obtained as orange solid (148 mg, 35 % (2 steps)). ^{1}H NMR (500 MHz, DMSO-d_6) δ ppm: 10.64 (s, 1H), 7.99 (d, *J* = 2.5 Hz, 1H), 7.60 (d, *J* = 8.5 Hz, 1H), 7.50 (dd, *J* = 2.5, 8.5 Hz, 1H), 4.58 (dd, *J* = 7.0, 8.5 Hz, 1H), 1.94–1.53 (m, 7H), 1.48–1.31 (m, 1H), 1.26–1.05 (m, 3H), 1.02–0.83 (m, 2H). ^{13}C NMR (126 MHz, DMSO-d_6) δ 167.5, 138.5, 131.2, 130.9, 125.6, 120.7, 119.6, 57.1, 41.1, 34.2, 32.6, 31.8, 25.9, 25.6, 25.5. HRMS (ESI$^+$) calculated for $C_{15}H_{19}Cl_3NO$ $[M+H]^+$ 334.05322, found 334.04984.

2-Chloro-3-cyclopropyl-*N*-(3',4'-dichlorophenyl)propanamide (5c).

Compound **5c** was synthesized in two steps. The first step was performed according to the **general procedure i**, using DL-3-cyclopropylalanine (500 mg, 3.87 mmol) and $NaNO_2$ (668 mg, 9.68 mmol). The obtained crude product **4c** was used in the next step without further purification. The second step was performed according to the **general procedure iii**, using 3,4-dichloroaniline (248 mg, 1.53 mmol), 2-chloro-3-cyclopropylpropanoic acid (273 mg, 1.84 mmol), $ClCO_2Et$ (200 μL, 2.03 mmol), Et_3N (260 μL, 1.84 mmol) and THF (20 mL). The reaction was stirred at room temperature overnight. The crude product was purified using column chromatography (Cyhex/EtOAc, 9:1). The product **5c** was obtained as yellow oil (121 mg, 11% (2 steps)). ^{1}H NMR (500 MHz, $CDCl_3$) δ ppm: 8.33 (*br* s, 1H), 7.81 (d, *J* = 2.3 Hz, 1H), 7.44–7.37 (m, 2H), 4.54 (t, *J* = 6.1 Hz, 1H), 2.02 (t, *J* = 6.5 Hz, 2H), 1.04–0.93 (m, 1H), 0.59–0.49 (m, 2H), 0.28–0.15 (m, 2H). ^{13}C NMR (126 MHz, $CDCl_3$) δ ppm: 167.0, 136.3, 132.9, 130.6, 128.3, 121.6, 119.1, 61.6, 40.4, 7.7, 4.74, 3.9. MS (ESI$^+$) *m/z* 291.94 $[M+H]^+$.

2-Chloro-*N*,3-diphenylpropanamide (5d)

Compound **5d** was prepared according to **general procedure ii**, using compound **4a** (934 mg 5.05 mmol), $SOCl_2$ (734 μL, 10.1 mmol) and aniline (507 μL, 5.56 mmol). Purification was done by flash chromatography (Hex/EtOAc, 100:0 to 0:100). The product was obtained as white solid (404 mg, 31%). ^{1}H NMR (500 MHz, DMSO-d_6) δ ppm: 7.95 (s, 1H), 7.60–7.52 (m, 2H), 7.38–7.28 (m, 6H), 7.27–7.19 (m, 1H), 7.11–7.04 (m, 1H), 4.76 (t, *J* = 7.5 Hz, 1H), 3.41 (dd, *J* = 13.8, 7.2 Hz, 1H), 3.13 (dd, *J* = 13.9, 7.8 Hz, 1H). MS (ESI$^+$) *m/z* 260.08 $[M+H]^+$.

2-Chloro-3-phenyl-*N*-(*o*-tolyl)propenamide (5e)

Compound **5e** was prepared according to **general procedure ii**, using compound **4a** (259 mg, 1.40 mmol), *o*-toluidine (165 mg, 1.54 mmol), $SOCl_2$ (203 μL, 2.80 mmol). The crude product was obtained as yellow oil (257 mg, 67%) and used in the next step without further purification. ^{1}H NMR (500 MHz, $CDCl_3$) δ ppm: 8.03 (s, 1H), 7.81 (d, *J* = 7.9 Hz, 1H), 7.35–7.25 (m, 5H), 7.23 (t, *J* = 7.7 Hz, 1H), 7.17 (d, *J* = 7.3 Hz, 1H), 7.10 (td, *J* = 7.5, 1.0 Hz, 1H), 4.75 (dd, *J* = 7.6, 4.3 Hz, 1H), 3.54 (dd, *J* = 14.3, 4.3 Hz, 1H), 3.35 (dd, *J* = 14.3, 7.6 Hz, 1H), 2.12 (s, 3H). ^{13}C NMR (126 MHz, $CDCl_3$) δ ppm: 166.2, 136.0, 134.9, 130.7, 130.0, 129.3, 128.6, 127.6, 127.0, 125.8, 122.6, 62.4, 41.6, 17.5. MS (ESI$^+$) *m/z* 274.09 [M+H]$^+$.

2-Chloro-3-phenyl-*N*-(*m*-tolyl)propenamide (5f)

Compound **5f** was prepared according to **general procedure ii**, using compound **4a** (775 mg, 4.19 mmol), *m*-toluidine (494 mg, 4.61 mmol), $SOCl_2$ (610 μL, 8.38 mmol). The product was purified using column chromatography (Hex/EtOAc, 100:0 to 0:100). The final product was obtained as yellow oil (510 mg, 44%). ^{1}H NMR (500 MHz, $CDCl_3$) δ ppm: 7.95 (s, 1H), 7.28–7.20 (m, 6H), 7.18–7.14 (m, 2H), 6.91 (d, *J* = 7.2 Hz, 1H), 4.60 (dd, *J* = 7.9, 4.4 Hz, 1H), 3.47 (dd, *J* = 14.3, 4.3 Hz, 1H), 3.23 (dd, *J* = 14.4, 7.9 Hz, 1H), 2.28 (s, 3H). MS (ESI$^+$) *m/z* 274.07 [M+H]$^+$.

2-Chloro-3-phenyl-*N*-(*p*-tolyl)propanamide (5g)

Compound **5g** was prepared according to **general procedure ii**, using compound **4a** (200 mg, 1.08 mmol), $SOCl_2$ (157 μL, 2.17 mmol) and *p*-toluidine (128 mg, 1.19 mmol). Purification was done by flash chromatography (Hex/EtOAc, 100:0 to 0:100). The final product was obtained as yellow solid (198 mg, 67%). ^{1}H NMR (500 MHz, $CDCl_3$) δ ppm: 8.02 (br s, 1H), 7.37–7.26 (m, 7H), 7.15 (d, *J* = 8.2 Hz, 2 H), 4.68 (dd, *J* = 7.8, 4.4 Hz, 1H), 3.54 (dd, *J* = 14.3, 4.4 Hz, 1H), 3.31 (dd, *J* = 14.3, 7.8 Hz, 1H), 2.34 (s, 3H). MS (ESI$^+$) *m/z* 274.09 [M+H]$^+$.

***S*-(1-((3,4-Dichlorophenyl)amino)-1-oxo-3-phenylpropan-2-yl) ethanethioate (6a)**

Compound **6a** was prepared according to **general procedure iv**, using compound **5a** (388 mg, 1.18 mmol) and potassium thioacetate (202 mg, 1.77 mmol) in acetone (10 mL). Purification was done by flash chromatography (Hex/EtOAc 100:0 to 0:100). The final product was obtained as colorless oil (361 mg, 83%). ^{1}H NMR (500 MHz, $CDCl_3$) δ ppm: 8.06 (s, 1H), 7.70 (d, *J* = 2.4 Hz, 1H), 7.31 (m, 3H), 7.26 (m, 4H), 4.26 (dd, *J* = 8.4, 7.2 Hz, 1H), 3.42 (dd, *J* = 14.1, 8.5 Hz, 1H), 2.99 (dd, *J* = 14.2, 7.0 Hz, 1H), 2.39 (s, 3H). ^{13}C NMR (126 MHz, $CDCl_3$)

δ ppm: 198.0, 168.7, 137.4, 137.2, 132.9, 130.6, 129.3, 128.8, 127.7, 127.3, 121.6, 119.1, 48.4, 35.6, 30.6. MS (ESI$^+$) *m/z* 369.11 $[M+H]^+$, 327.11 $[M–Ac+2H]^+$.

2-*S*-(Acetylthio)-*N*-(3’,4’-dichlorophenyl)-3-cyclohexylpentanamide (6b).

Compound **6b** was synthesized according to **general procedure iv**, using compound **5b** (100 mg, 0.299 mmol), potassium thioacetate (68 mg, 0.60 mmol) and acetone (5 mL). The reaction was stirred at room temperature overnight. The crude product was purified using column chromatography (100% CH_2Cl_2). Compound **6b** was obtained as yellow oil (49 mg, 44%). ^{1}H NMR (500 MHz, DMSO-d_6) δ ppm: 10.59 (s, 1H), 7.98 (*br* s, 1H), 7.57 (d, *J* = 8.5 Hz, 1H), 7.50 (*br* d, *J* = 9.0 Hz, 1H), 4.26 (*br* t, *J* = 7.8 Hz, 1H), 2.36 (s, 3H), 1.87–1.75 (m, 2H), 1.70–1.44 (m, 5H), 1.30–1.04 (m, 4H), 0.99–0.82 (m, 2H). ^{13}C NMR (126 MHz, DMSO-d_6) δ ppm: 194.3, 169.6, 138.8, 131.1, 130.8, 125.2, 120.6, 119.5, 45.8, 39.5, 35.1, 32.5, 32.3, 30.3, 25.9, 25.6, 25.5. HRMS (ESI$^+$) calculated for $C_{17}H_{22}Cl_2NO_2S$ $[M+H]^+$ 374.07483, found 374.07126.

2-*S*-(Acetylthio)-*N*-(3’,4’-dichlorophenyl)-3-cyclopropylpentanamide (6c).

Compound **6c** was synthesized according to the **general procedure iv**, using compound **5c** (118 mg, 0.400 mmol), potassium thioacetate (92 mg, 0.81 mmol) and acetone (4 mL). The reaction was stirred at room temperature overnight. The crude product was purified using column chromatography (Cyhex/EtOAc, 8:2). Compound **6c** was obtained as yellow oil (88 mg, 66%). ^{1}H NMR (500 MHz, $CDCl_3$) δ ppm: 8.26 (*br* s, 1H), 7.76 (*br* s, 1H), 7.37–7.30 (m, 2H), 4.10 (t, *J* = 7.5 Hz, 1H), 2.41 (s, 3H), 2.07–1.98 (m, 1H), 1.57–1.53 (m, 1H), 0.88–0.78 (m, 1H), 0.54–0.45 (m, 2H), 0.19–0.12 (m, 2H). ^{13}C NMR (126 MHz, $CDCl_3$) δ 198.3, 169.2, 137.2, 132.8, 130.4, 127.4, 121.3, 118.9, 47.2, 34.4, 30.4, 9.0, 4.8, 4.7. MS (ESI$^+$) *m/z* 331.98 $[M+H]^+$.

***S*-(1-Oxo-3-phenyl-1-(phenylamino)propan-2-yl) ethanethioate (6d)**

Compound **6d** was prepared according to **general procedure iv**, using compound **5d** (242 mg, 0.93 mmol) and potassium thioacetate (118 mg, 1.12 mmol) in acetone (10 mL). Purification was done by flash chromatography (Hex/EtOAc, 100:0 to 0:100). The final product was obtained as colorless oil (127 mg, 46%). ^{1}H NMR (500 MHz, $CDCl_3$) δ ppm: 7.96 (br s, 1H), 7.46 (d, *J* = 8.2 Hz, 2H), 7.33–7.22 (m, 5H), 7.12–7.07 (m, 2H), 7.09 (t, *J* = 7.4 Hz, 1H), 4.30 (t, *J* = 7.7 Hz, 1H), 3.46 (dd, *J* = 14.1, 8.5 Hz, 1H), 3.01 (dd, *J* = 14.1, 7.1 Hz, 1H), 2.38 (s,

3H). ^{13}C NMR (126 MHz, $CDCl_3$) δ ppm: 197.3, 168.3, 137.6, 137.6, 129.2, 128.9, 128.6, 127.0, 124.4, 119.8, 48.5, 35.7, 30.4. MS (ESI$^+$) *m/z* 300.17 $[M+H]^+$, 258.10 $[M–Ac+2H]^+$.

***S*-(1-Oxo-3-phenyl-1-(*o*-tolylamino)propan-2-yl) ethanethioate (6e)**

Compound **6e** was prepared according to **general procedure iv**, using compound **5e** (257 mg, 0.94 mmol) and potassium thioacetate (161 mg, 1.41 mmol) in acetone (10 mL). Purification was done by flash chromatography (Hex/EtOAc, 9:1). The final product was obtained as white solid (149 mg, 51%). ^{1}H NMR (500 MHz, $CDCl_3$) δ ppm: 7.85 (d, *J* = 8.2 Hz, 1H), 7.73 (s, 1H), 7.34–7.26 (m, 4H), 7.23–7.10 (m, 3H), 7.04 (m, 1H), 4.34 (dd, *J* = 8.5, 7.0 Hz, 1H), 3.54–3.38 (m, 1H), 3.03 (dd, *J* = 14.1, 7.0 Hz, 1H), 2.38 (s, 3H), 2.14 (s, 3H). ^{13}C NMR (126 MHz, $CDCl_3$) δ ppm: 197.1, 168.5, 137.8, 135.8, 130.5, 129.4, 128.7, 127.1, 126.8, 125.1, 122.5, 48.5, 35.9, 30.5, 17.7. MS (ESI$^+$) *m/z* 314.17 $[M+H]^+$, 272.06 $[M–Ac+2H]^+$.

***S*-(1-Oxo-3-phenyl-1-(*m*-tolylamino)propan-2-yl) ethanethioate (6f)**

Compound **6f** was prepared according to **general procedure iv**, using compound **5f** (510 mg, 1.86 mmol) and potassium thioacetate (319 mg, 2.79 mmol) in acetone (10 mL). Purification was done by flash chromatography (Hex/EtOAc, 7:3). The final product was obtained as white powder (300 mg, 51%). ^{1}H NMR (500 MHz, $CDCl_3$) δ 7.91 (s, 1H), 7.31–7.25 (m, 3H), 7.24–7.18 (m, 4H), 7.18–7.12 (m, 1H), 6.88 (d, *J* = 7.5 Hz, 1H), 4.26 (dd, *J* = 8.4, 7.2 Hz, 1H), 3.42 (dd, *J* = 14.1, 8.4 Hz, 1H), 2.97 (dd, *J* = 14.1, 7.1 Hz, 1H), 2.34 (s, 3H), 2.29 (s, 3H). ^{13}C NMR (126 MHz, $CDCl_3$) δ ppm: 197.5, 168.4, 139.0, 137.7, 137.6, 129.3, 128.9, 128.7, 127.1, 125.4, 120.5, 116.9, 48.6, 35.8, 30.5, 21.6. MS (ESI$^+$) *m/z* 314.06 $[M+H]^+$, 271.99 $[M–Ac+2H]^+$.

***S*-(1-Oxo-3-phenyl-1-(*p*-tolylamino)propan-2-yl) ethanethioate (6g)**

Compound **6g** was prepared according to **general procedure iv**, using compound **5g** (190 mg, 0.69 mmol) and potassium thioacetate (119 mg, 1.04 mmol). Purification was done by flash chromatography (Hex/EtOAc, 100:0 to 0:100). The product was obtained as yellow solid (156 mg, 72%). ^{1}H NMR (500 MHz, $CDCl_3$) δ ppm: 7.87 (d, *J* = 11.9 Hz, 1H), 7.32–7.26 (m, 5H), 7.26–7.15 (m, 2H), 7.10 (d, *J* = 8.2 Hz, 2H), 4.32–4.26 (m, 1H), 3.45 (dd, *J* = 14.2, 8.4 Hz, 1H), 3.01 (dd, *J* = 14.0, 7.0 Hz, 1H), 2.37 (s, 3H), 2.31 (s, 3H). ^{13}C NMR (126 MHz, $CDCl_3$) δ ppm: 197.2, 168.1, 137.6, 135.0, 134.1, 129.4, 129.2, 128.6, 127.0, 119.8, 48.5, 35.8, 30.4, 20.8. MS (ESI$^+$) *m/z* 314.10 $[M+H]^+$, 272.04 $[M–Ac+2H]^+$.

***N*-(3,4-Dichlorophenyl)-2-mercapto-3-phenylpropanamide (7a)**

Compound **7a** was prepared according to **general procedure v**, using compound **6a** (361 mg, 0.98 mmol) and 2 M NaOH aq. solution (980 μL, 1.96 mmol). Purification was done by chromatography (Hex/EtOAc, 100:0 to 0:100). The final product was obtained as white solid (138 mg, 43%). ^{1}H NMR (500 MHz, $CDCl_3$) δ ppm: 8.06 (br s, 1H), 7.71 (d, *J* = 2.0 Hz, 1H), 7.37 (d, *J* = 8.7 Hz, 1H), 7.31 (dd, *J* = 14.6, 6.9 Hz, 3H), 7.28–7.25 (m, 1H), 7.22 (d, *J* = 7.2 Hz, 2H), 3.72 (dd, *J* = 15.3, 6.9 Hz, 1H), 3.34 (dd, *J* = 14.3, 6.6 Hz, 1H), 3.25 (dd, *J* = 13.9, 6.7 Hz, 1H), 2.11 (d, *J*=9.0 Hz, 1H). ^{13}C NMR (126 MHz, $CDCl_3$) δ ppm: 169.8, 137.1, 136.8, 133.0, 130.7, 129.6, 128.8, 128.2, 127.4, 121.8, 119.3, 45.9, 41.5. HRMS (ESI^-) m/z calcd. for $C_{15}H_{12}Cl_2NOS$ $[M–H]^-$ 324.00221, found 324.00235.

2-Mercapto-*N*-(3',4'-dichlorophenyl)-3-cyclohexylpropanamide (7b).

Compound **7b** was synthesized according to the **general procedure v**, using compound **6b** (40 mg, 0.11 mmol), sodium hydroxide (13 mg, 0.32 mmol) and MeOH (5 mL). The reaction was stirred at room temperature for 3 h. The crude product was purified using preparative HPLC (CH_3CN (FA 0.05 %)/H_2O (FA 0.05 %) 5:95 → 90:10). The product was obtained as colorless oil (11 mg, 31%). ^{1}H NMR (500 MHz, DMSO-d_6) δ ppm: 10.39 (s, 1H), 8.00 (d, *J* = 2.5 Hz, 1H), 7.57 (d, *J* = 8.5 Hz, 1H), 7.48 (dd, *J* = 2.5, 8.5 Hz, 1H), 3.53 (dd, *J* = 7.0, 8.0 Hz, 1H), 3.10 (*br* s, 1H), 1.88–1.45 (m, 7H), 1.36–1.03 (m, 4H), 0.96–0.79 (m, 2H). ^{13}C NMR (126 MHz, DMSO-d_6) δ ppm: 171.8, 139.1, 131.1, 130.8, 124.9, 120.4, 119.3, 42.5, 39.2, 35.1, 32.4, 32.4, 26.0, 25.7, 25.6. HRMS (ESI^+) calculated for $C_{15}H_{20}Cl_2NOS$ $[M+H]^+$ 332.06427, found 332.06421.

2-Mercapto-*N*-(3',4'-dichlorophenyl)-3-cyclopropylpropanamide (7c).

Compound **7c** was synthesized according to the **general procedure v**, using compound **6c** (88 mg, 0.26 mmol), sodium hydroxide (31 mg, 0.78 mmol) and 5 mL of MeOH. The reaction was stirred at room temperature for 2 h. After extraction, the product was purified column chromatography (Cyhex/EtOAc 8:2). The product was obtained as white solid (20 mg, 26%). ^{1}H NMR (500 MHz, $CDCl_3$) δ ppm: 8.21 (*br* s, 1H), 7.68 (s, 1H), 7.13 (s, 2H), 3.44 (q, *J* = 7.12 Hz, 1H), 2.03 (d, *J* = 8.54 Hz, 1H), 1.83–1.68 (m, 2H), 0.80–0.70 (m, 1H), 0.38 (*br* d, *J* = 7.93 Hz, 2H), 0.04 (*br* d, *J* = 4.73 Hz, 2H). ^{13}C NMR (126 MHz, $CDCl_3$) δ ppm: 170.5, 137.1, 133.1, 130.7, 128.0, 121.6, 119.1, 45.0, 40.5, 8.9, 4.7. HRMS (ESI^-) calculated for $C_{12}H_{12}Cl_2NOS$ $[M–H]^-$ 288.00221, found 288.00068.

2-Mercapto-*N*,3-diphenylpropanamide (7d)

Compound **7d** was prepared according to **general procedure v**, using compound **6d** (127 mg, 0.42 mmol) and 2 M NaOH aq. solution (420 μL, 0.84 mmol). Purification was done by flash chromatography (Hex/EtOAc 100:0 to 0:100). The final product was obtained as white solid (46 mg, 43%). ^{1}H NMR (500 MHz, $CDCl_3$) δ ppm: 8.02 (br s, 1H), 7.46 (d, *J* = 8.1 Hz, 2H), 7.36–7.29 (m, 4H), 7.29–7.23 (m, 3H), 7.14 (t, *J* = 7.6 Hz, 1H), 3.72 (dd, *J* = 14.8, 6.6 Hz, 1H), 3.38 (dd, *J* = 13.8, 6.5 Hz, 1H), 3.24 (dd, *J* = 13.8, 6.8 Hz, 1H), 2.11 (d, *J* = 8.9 Hz, 1H). ^{13}C NMR (126 MHz, $CDCl_3$) δ ppm: 169.5. 137.2, 137.1, 129.3, 129.0, 128.5, 127.1, 124.7, 120.0, 45.7, 41.3. HRMS (ESI^+) m/z calcd. for $C_{15}H_{16}NOS$ $[M+H]^+$ 258.09471, found 258.09434.

2-Mercapto-3-phenyl-*N*-(*o*-tolyl)propenamide (7e)

Compound **7e** was prepared according to **general procedure v**, using compound **6e** (148 mg, 0.47 mmol) and 2 M NaOH aq. solution (475 μL, 0.95 mmol) in MeOH (2.5 mL). Purification was done by column chromatography (Hex/EtOAc 7/3). The final product was obtained as white solid (25 mg, 20%). ^{1}H NMR (500 MHz, $CDCl_3$) δ 8.03 (s, 1H), 7.81 (d, *J* = 8.0 Hz, 1H), 7.38–7.24 (m, 5H), 7.22 (t, *J* = 7.8 Hz, 1H), 7.16 (d, *J* = 7.3 Hz, 1H), 7.08 (t, *J* = 7.2 Hz, 1H), 3.79 (dt, *J* = 8.8, 6.6 Hz, 1H), 3.36 (dd, *J* = 13.8, 6.4 Hz, 1H), 3.29 (dd, *J* = 13.8, 6.7 Hz, 1H), 2.11 (t, *J* = 4.4 Hz, 3H), 2.11 (d, *J* = 8.7 Hz, 1H). ^{13}C NMR (126 MHz, $CDCl_3$) δ ppm: 169.6, 137.4, 135.3, 130.6, 129.7, 129.1, 128.7, 127.3, 126.9, 125.5, 122.5, 46.1, 41.6, 17.7. HRMS (ESI^+) m/z calcd. for $C_{16}H_{18}NOS$ $[M+H]^+$ 272.11036, found 272.11025.

2-Mercapto-3-phenyl-*N*-(*m*-tolyl)propenamide (7f).

Compound **7f** was prepared according to **general procedure v**, using compound **6f** (298 mg, 0.95 mmol) and 2 M NaOH aq. solution (951 μL, 2 mmol) in MeOH (5 mL). The final product was obtained as white solid without further purification (227 mg, 88%).^{1}H NMR (500 MHz, Acetone-d_6) δ ppm: 9.17 (s, 1H), 7.43 (s, 1H), 7.37 (d, *J* = 8.1 Hz, 1H), 7.30–7.17 (m, 5H), 7.15 (t, *J* = 7.8 Hz, 1H), 6.87 (d, *J* = 7.5 Hz, 1H), 3.76–3.71 (m,1H), 3.38–3.29 (m, 1H), 3.00 (dd, *J* = 13.6, 6.3 Hz, 1H), 2.54 (d, *J* = 9.6 Hz, 1H), 2.27 (s, 3H). ^{13}C NMR (126 MHz, Acetone-d_6) δ ppm: 171.1, 139.9, 139.7, 139.1, 130.0, 129.4, 129.1, 127.4, 125.2, 120.7, 117.3, 45.1, 43.0, 21.4. HRMS (ESI^-) m/z calcd. for $C_{16}H_{16}NOS$ $[M-H]^-$ 270.09580, found 270.09560.

2-Mercapto-3-phenyl-*N*-(*p*-tolyl)propenamide (7g).

Compound **7g** was prepared according to **general procedure v**, using compound **6g** (100 mg, 0.32 mmol) and 2 M NaOH aq. solution (320 μL, 0.64 mmol). Purification was done by flash chromatography (Hex/EtOAc, 100:0 to 0:100). The final product was obtained as white solid (58 mg, 67%). ^{1}H NMR (500 MHz, $CDCl_3$) δ ppm: 7.94 (br s, 1H), 7.36–7.29 (m, 4H), 7.28–

7.23 (m, 3H), 7.13 (d, *J* = 8.4 Hz, 2H), 3.70 (dt, *J* = 8.8, 6.7 Hz, 1H), 3.37 (dd, *J* = 14.0, 6.4 Hz, 1H), 3.24 (dd, *J* = 13.0, 6.4 Hz, 1H), 2.31 (s, 3H), 2.10 (d, *J* = 8.9 Hz, 1H). ^{13}C NMR (126 MHz, $CDCl_3$) δ ppm: 169.5, 137.5, 134.8, 134.6, 129.7, 129.6, 128.7, 127.3, 120.2, 46.0, 41.7, 21.0. HRMS (ESI$^+$) m/z calcd. for $C_{16}H_{18}NOS$ $[M+H]^+$ 272.11036, found 272.1099.

NMR and LC-MS spectra of final compounds

^{1}H NMR Spectra of Compound 7a

CAB AV4 500 MHZ
1H
CDCl3

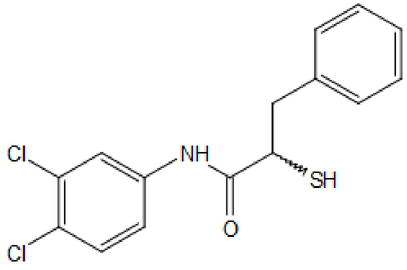

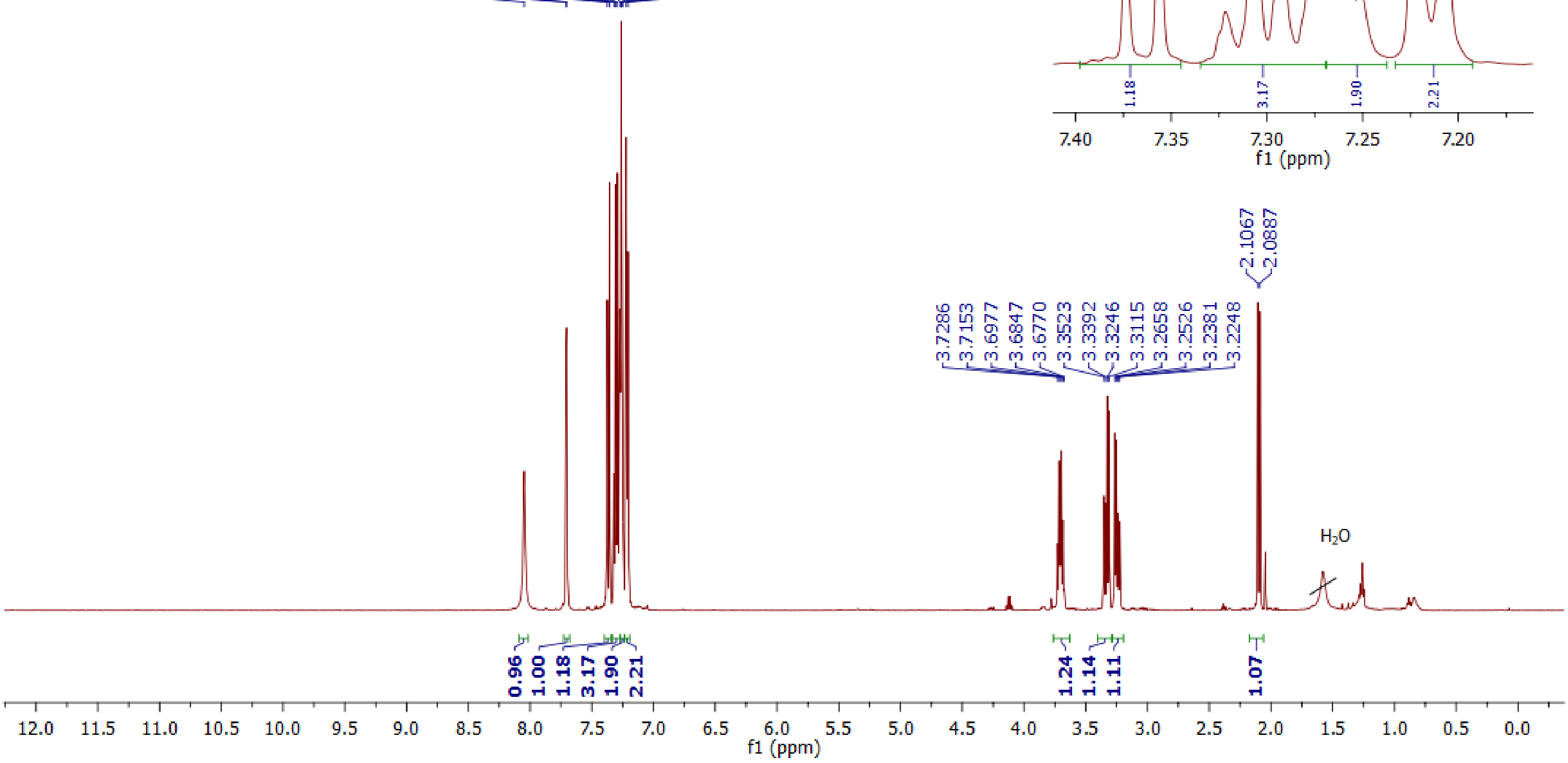

^{13}C NMR Spectra of Compound 7a

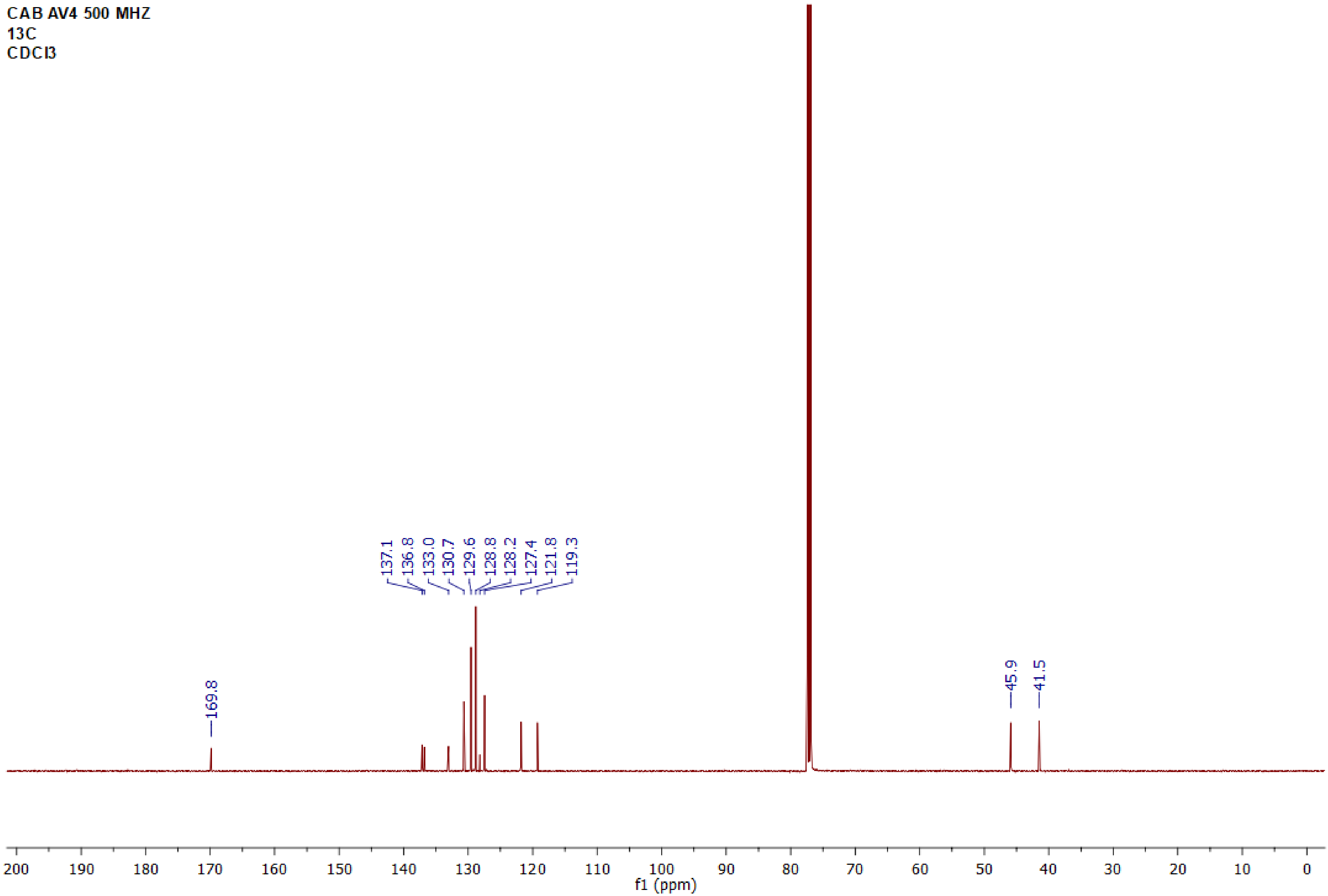

^{1}H NMR Spectra of Compound 7b

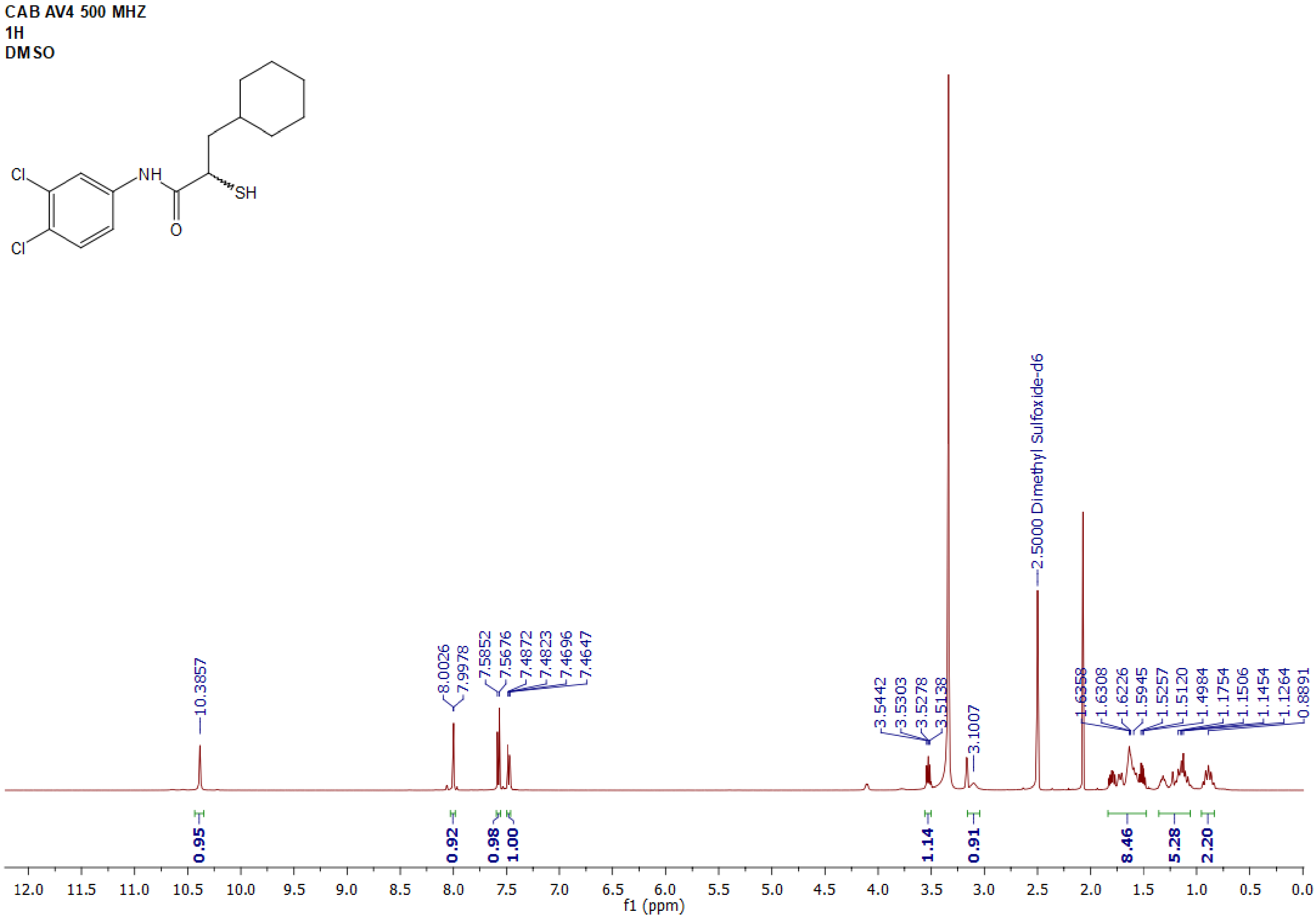

^{13}C NMR Spectra of Compound 7b

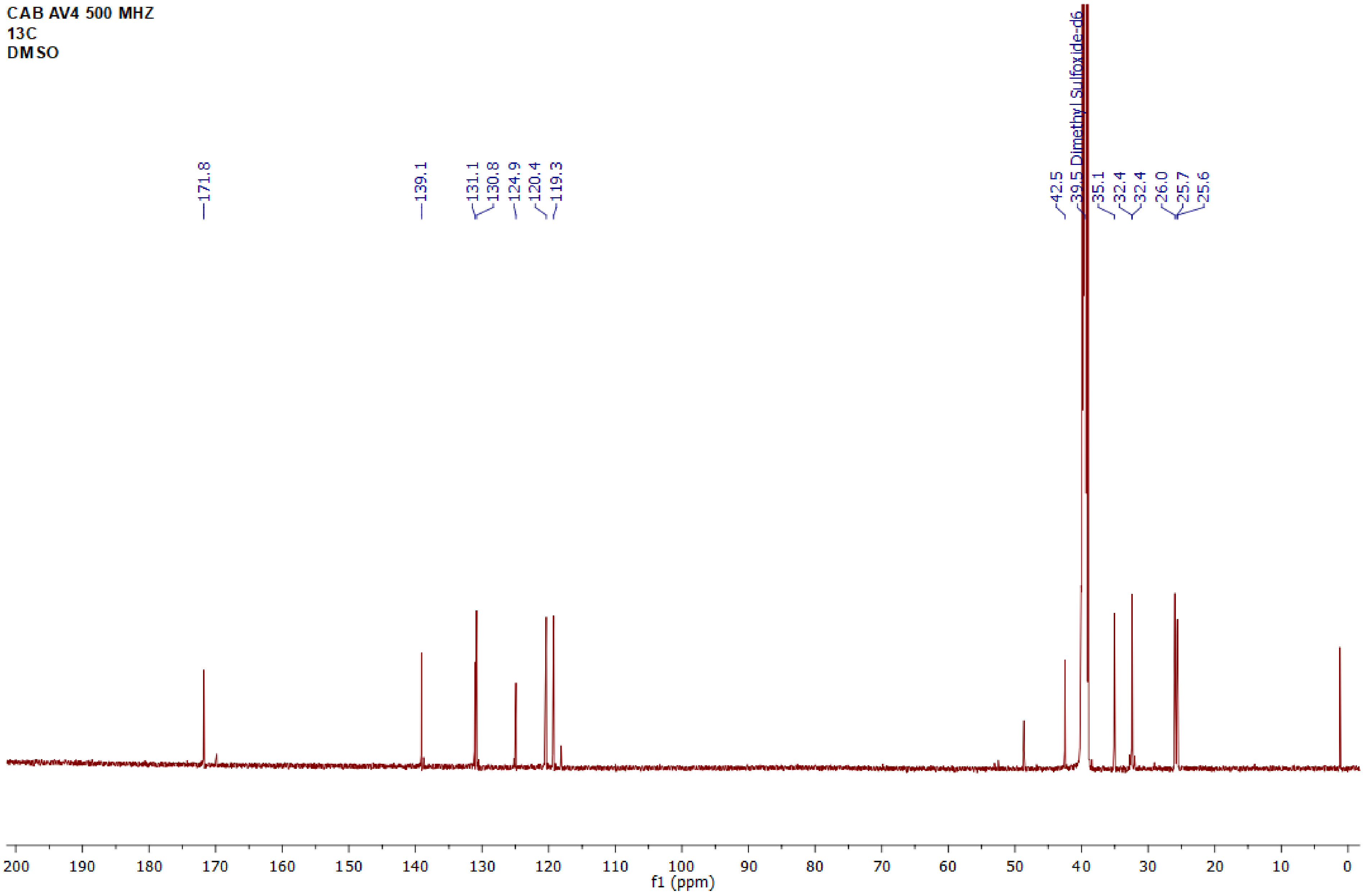

^{1}H NMR Spectra of Compound 7c

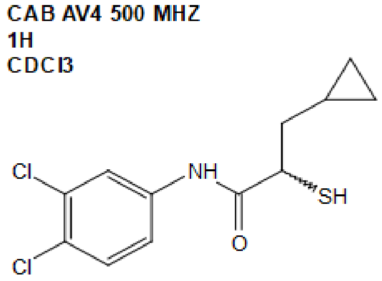

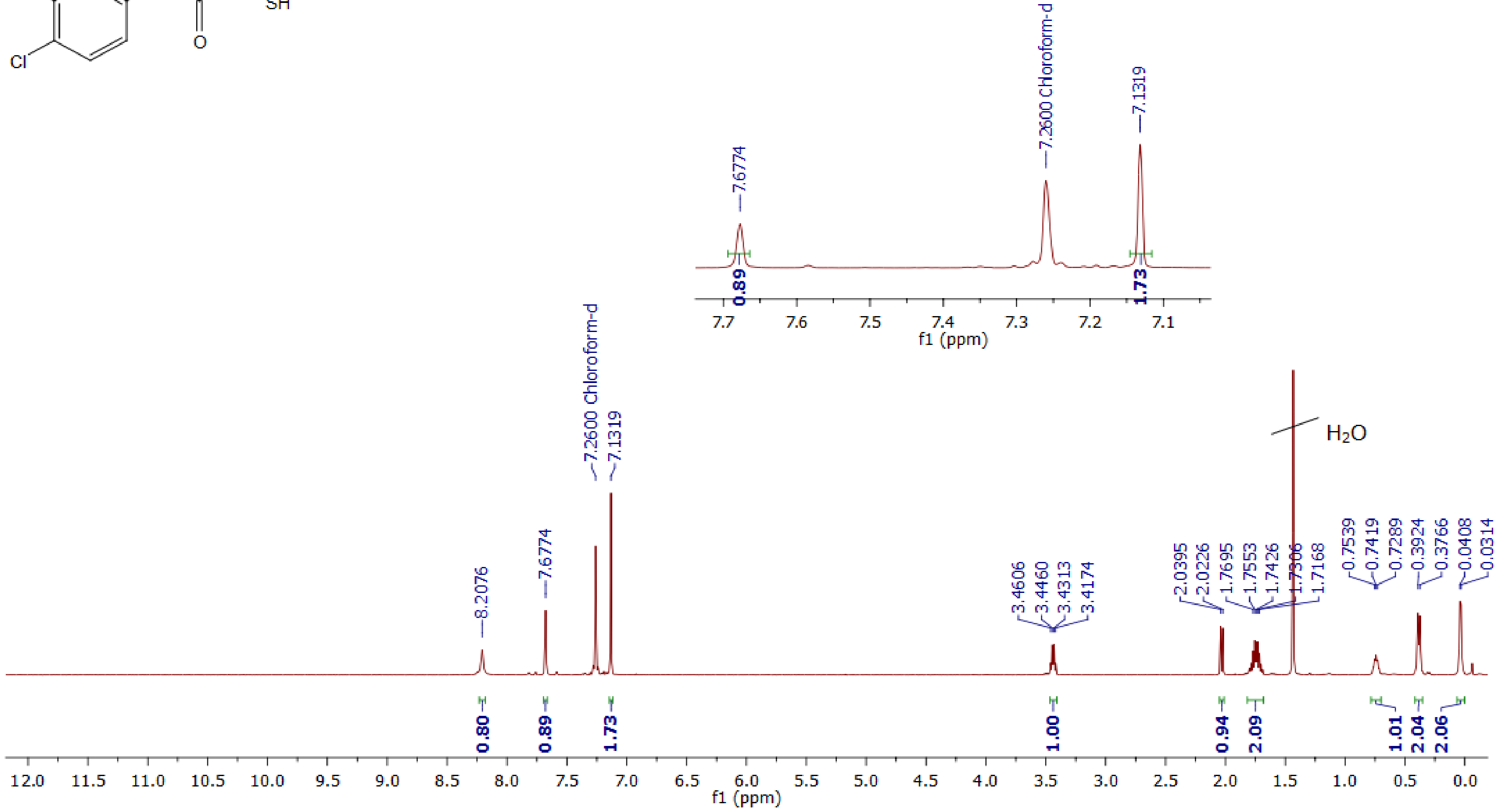

^{13}C NMR Spectra of Compound 7c

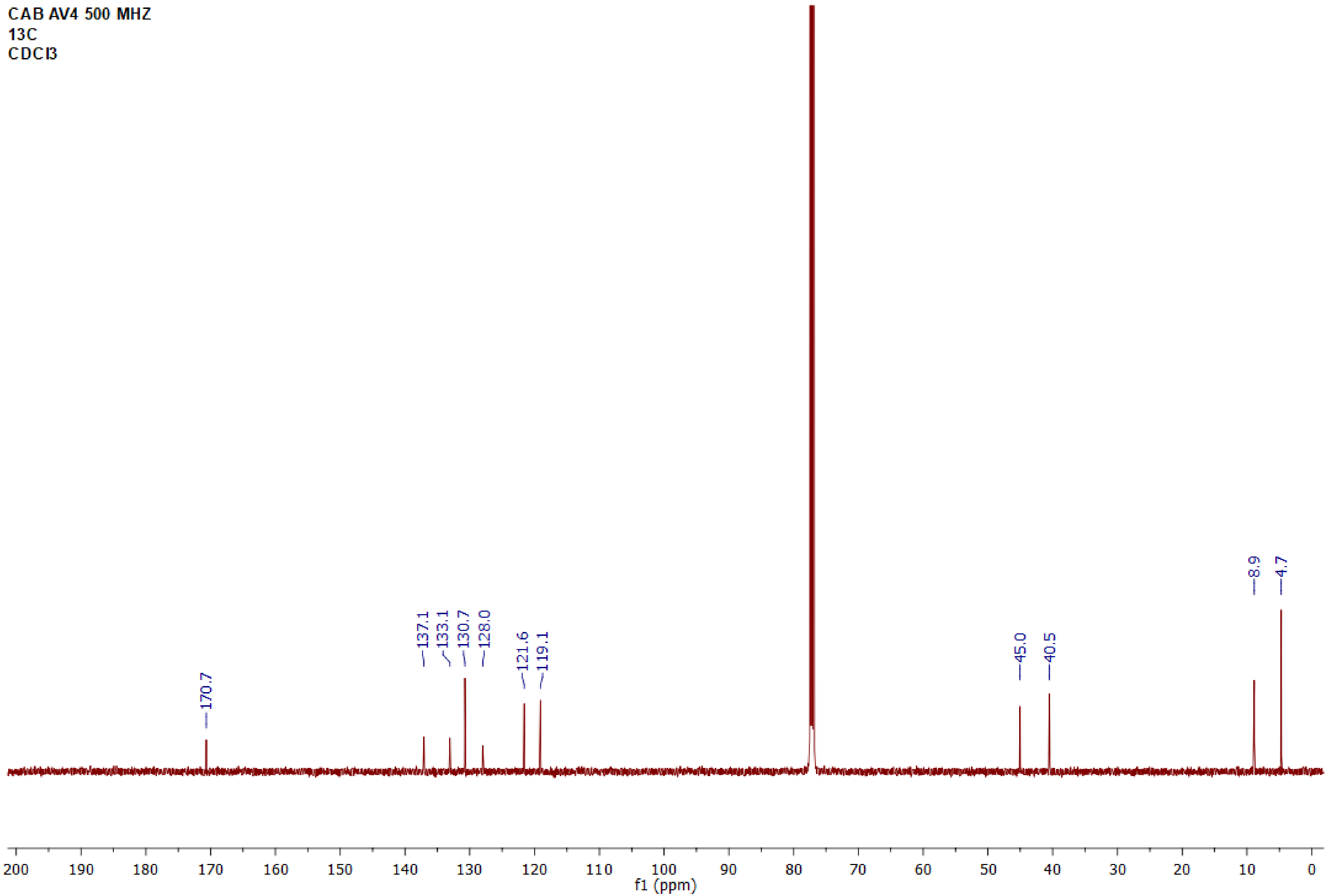

^{1}H NMR Spectra of Compound 7d

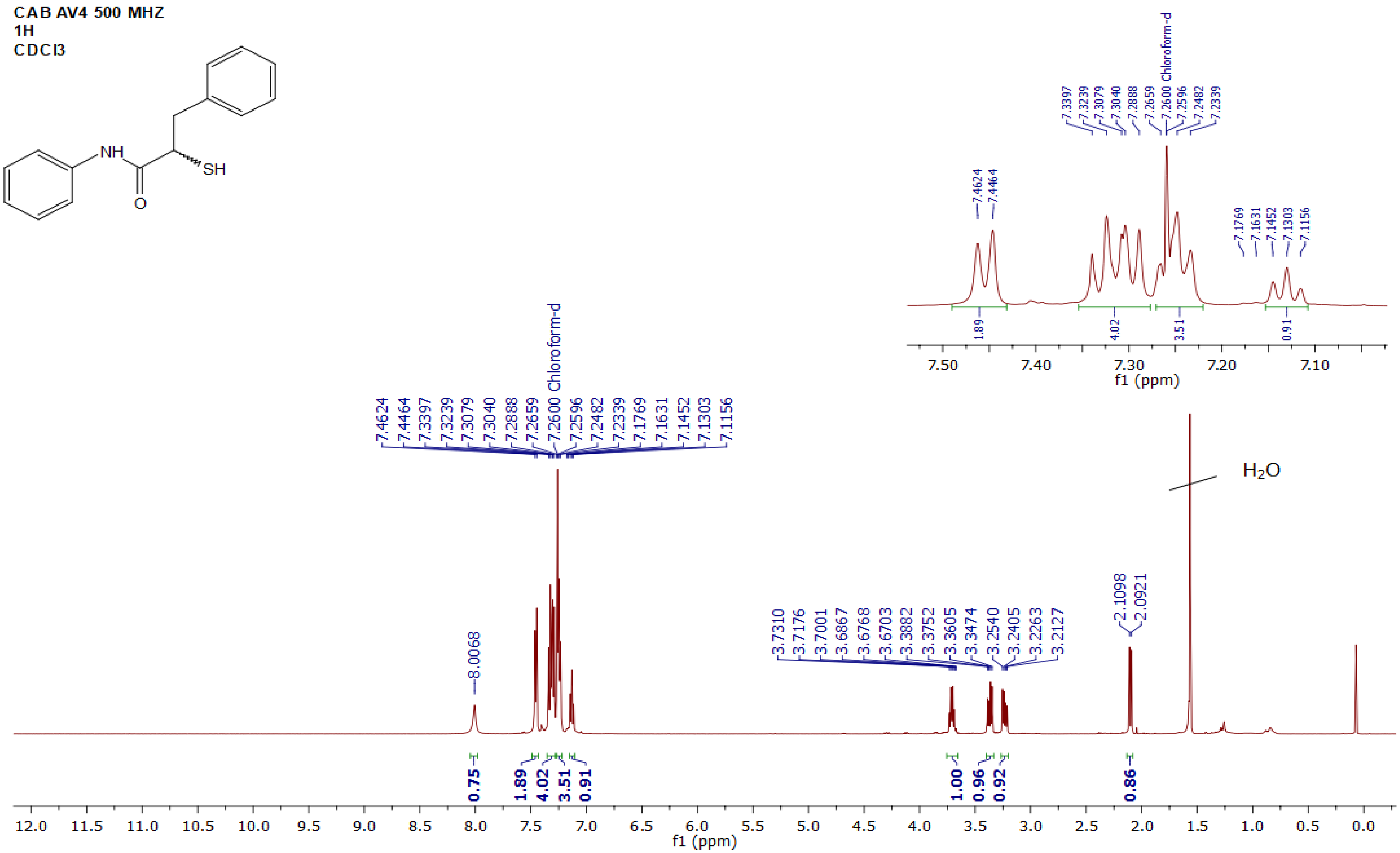

^{13}C NMR Spectra of Compound 7d

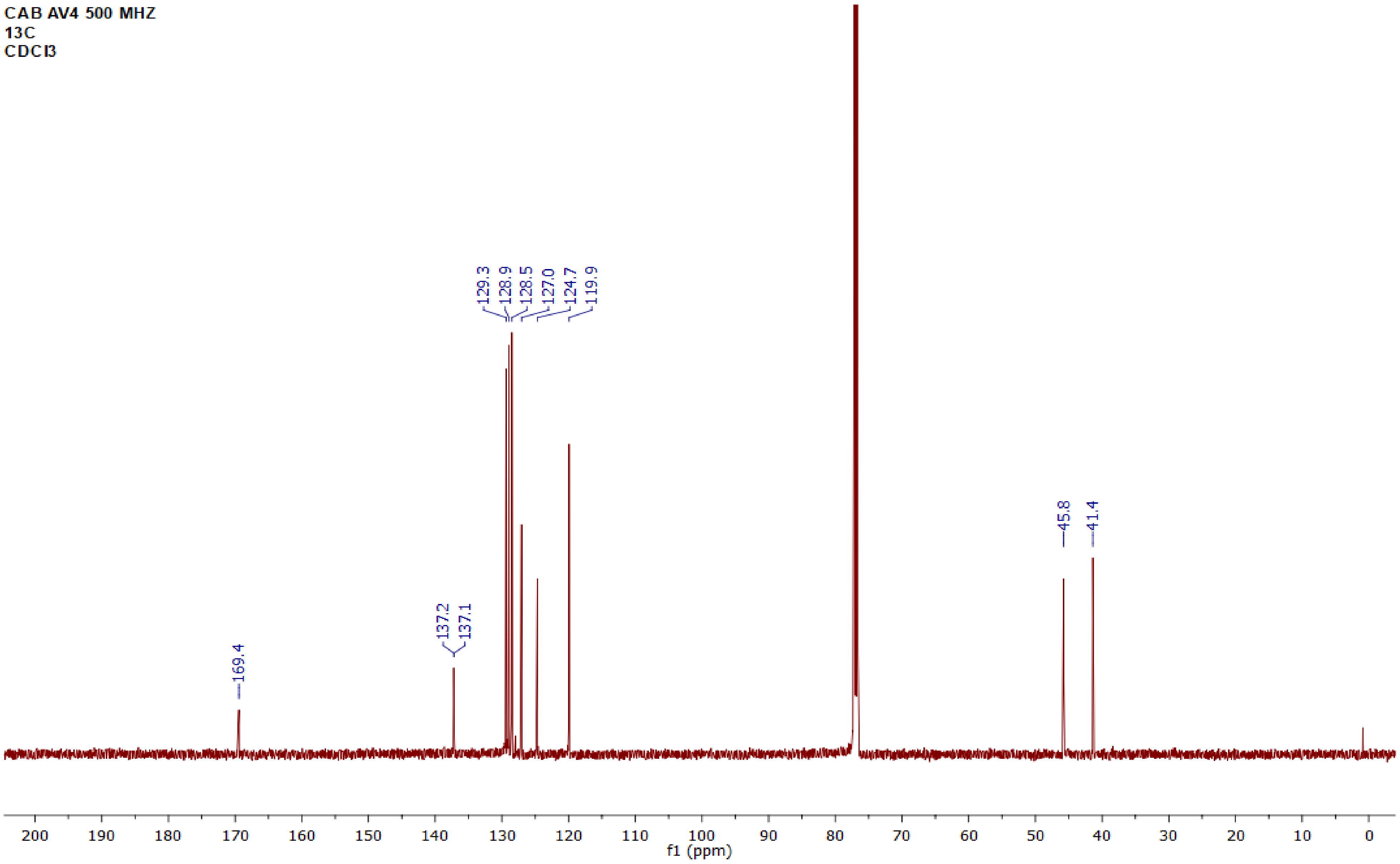

^{1}H NMR Spectra of Compound 7e

CAB AV4 500 MHZ
1H
CDCl3

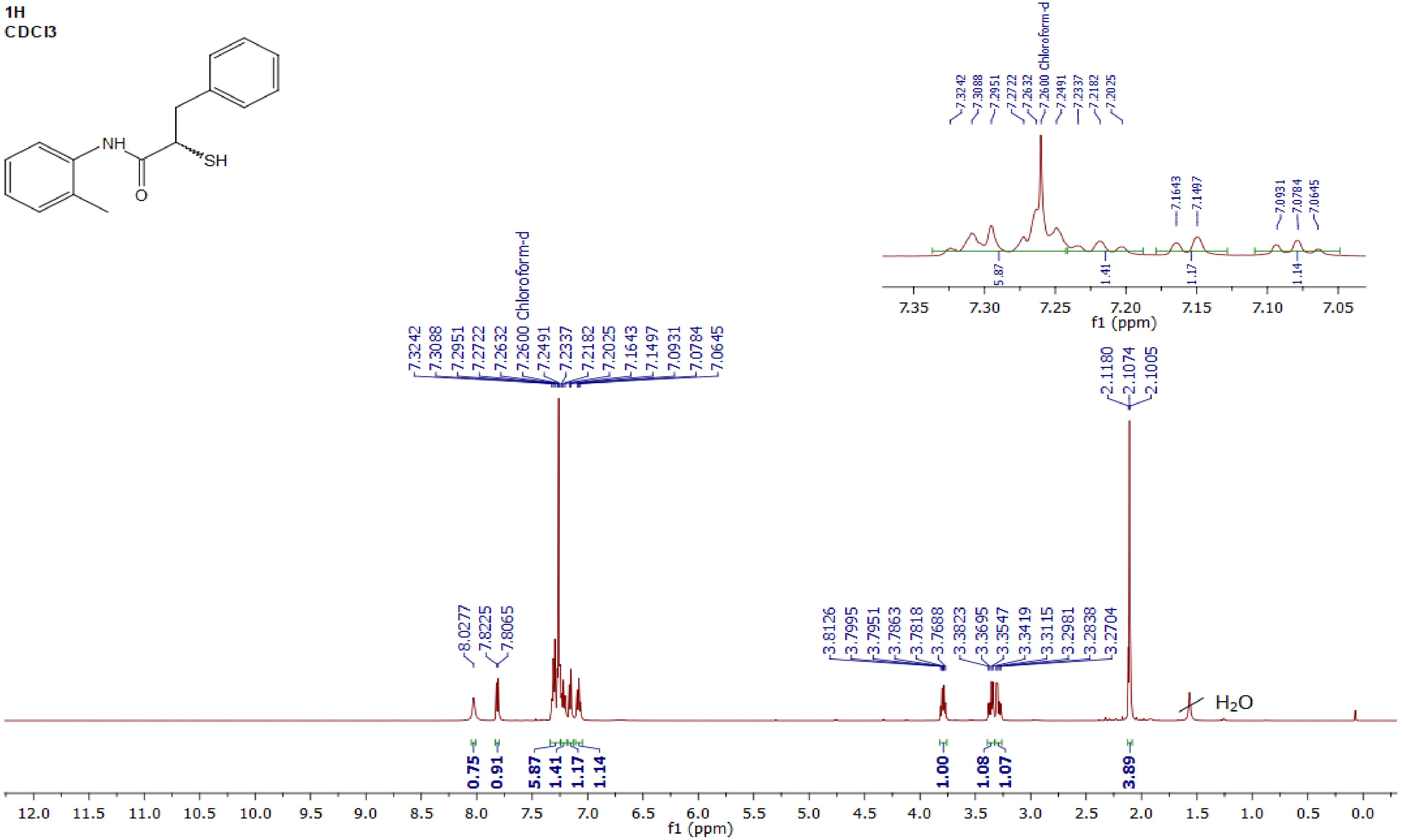

^{13}C NMR Spectra of Compound 7e

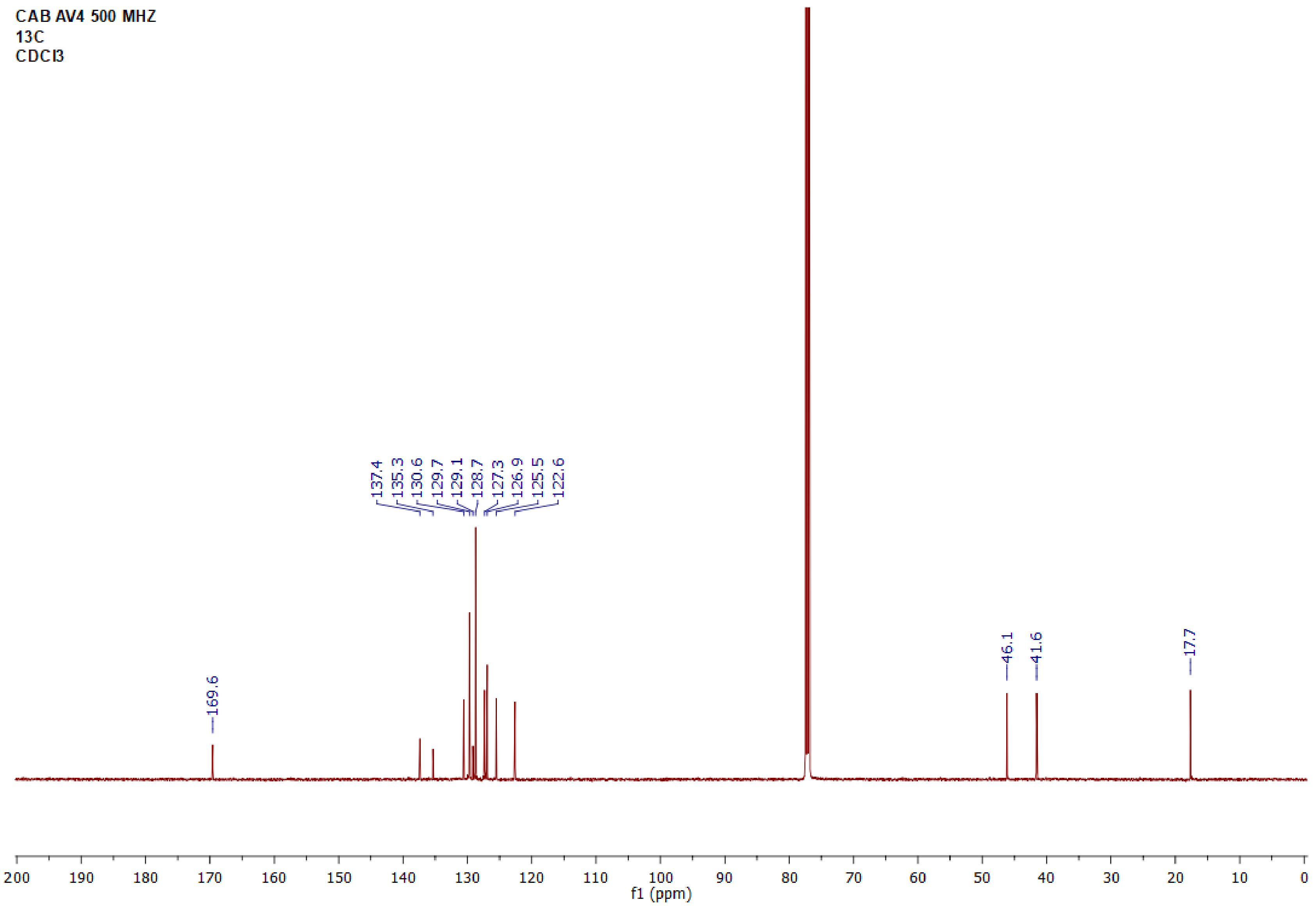

^{1}H NMR Spectra of Compound 7f

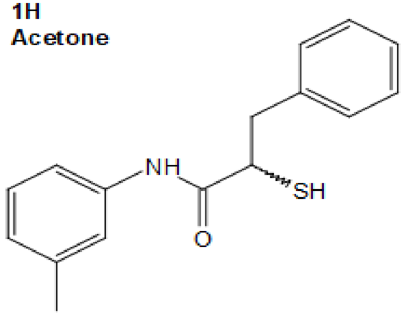

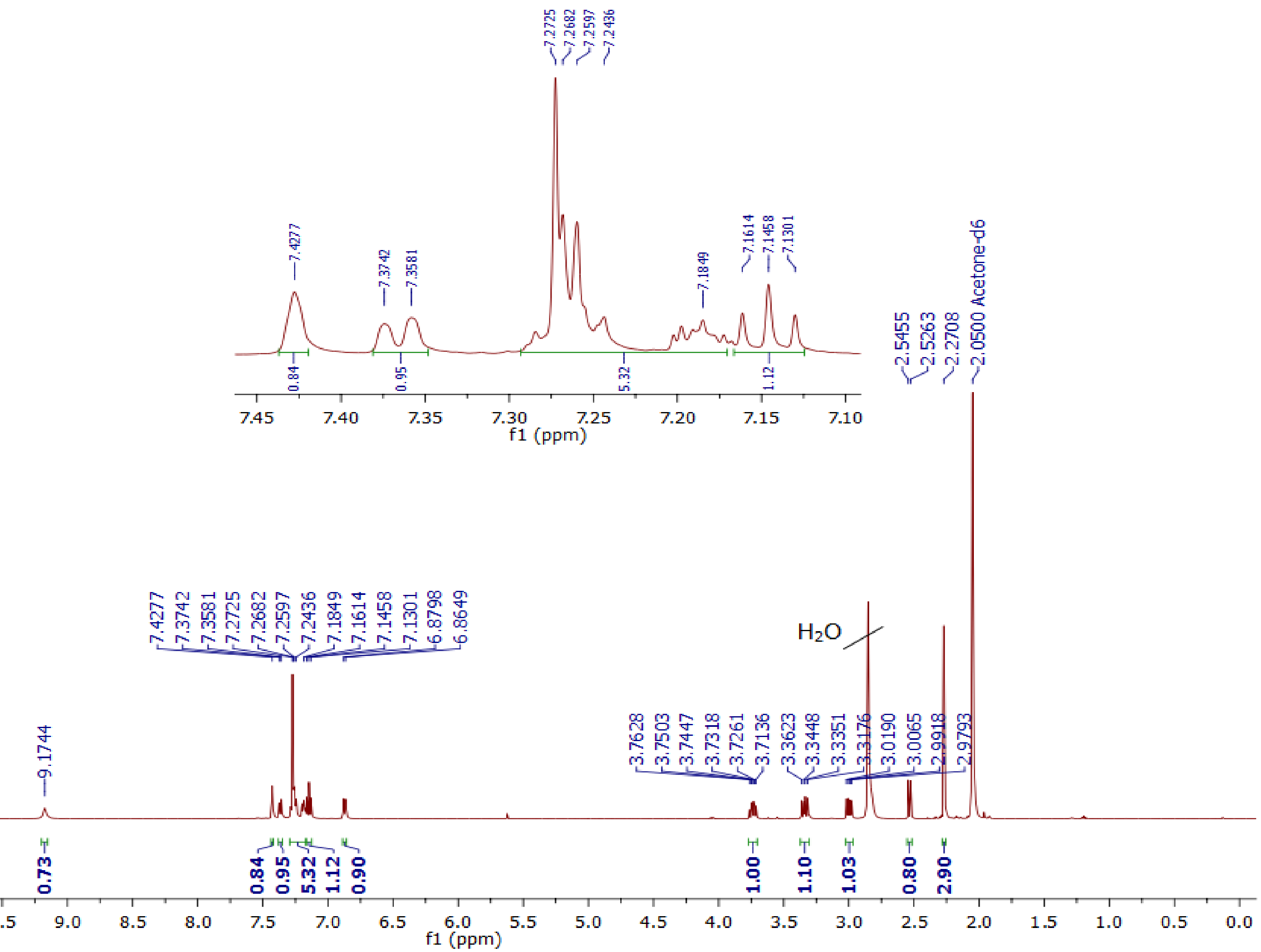

^{13}C NMR Spectra of Compound 7f

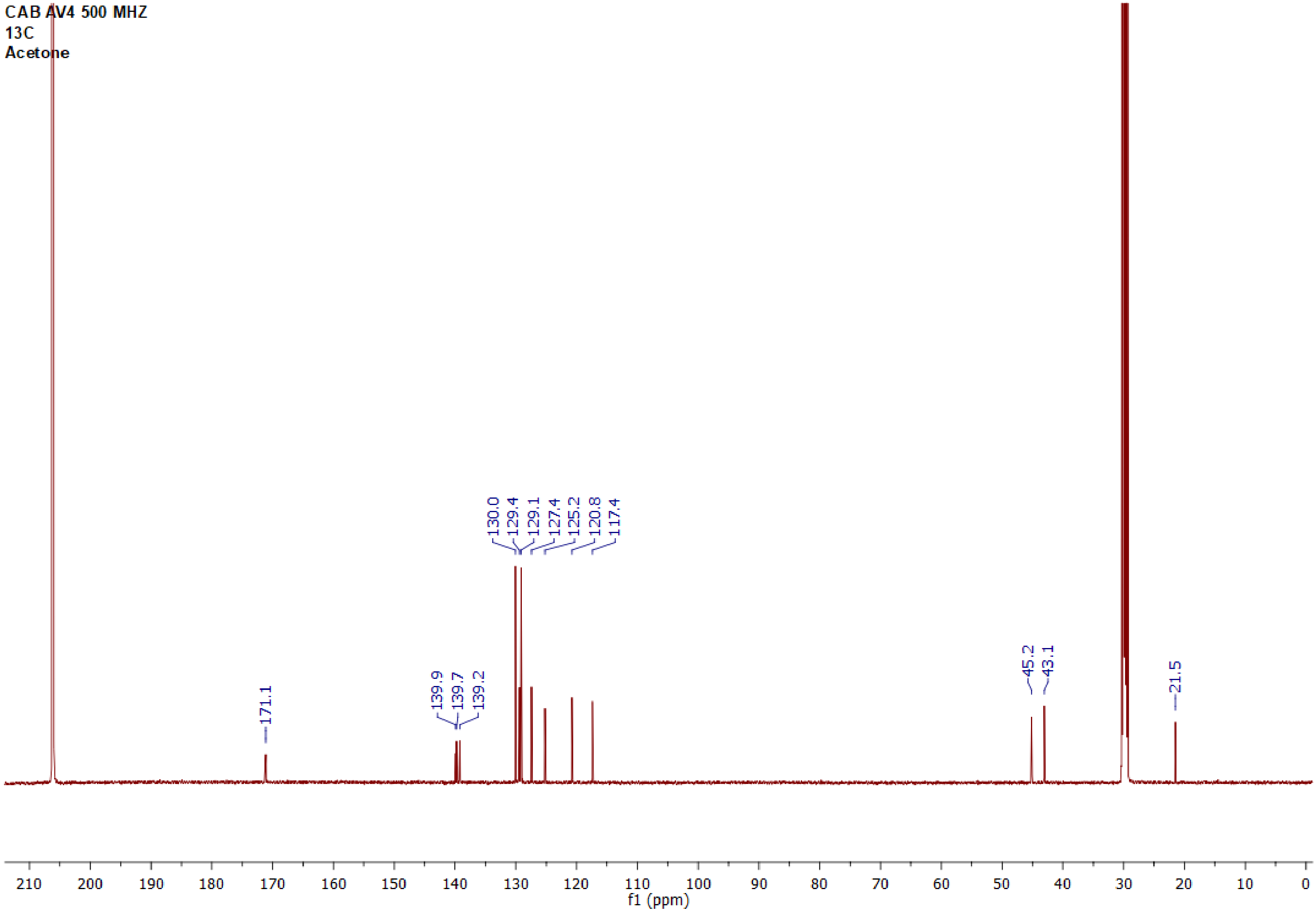

^{1}H NMR Spectra of Compound 7g

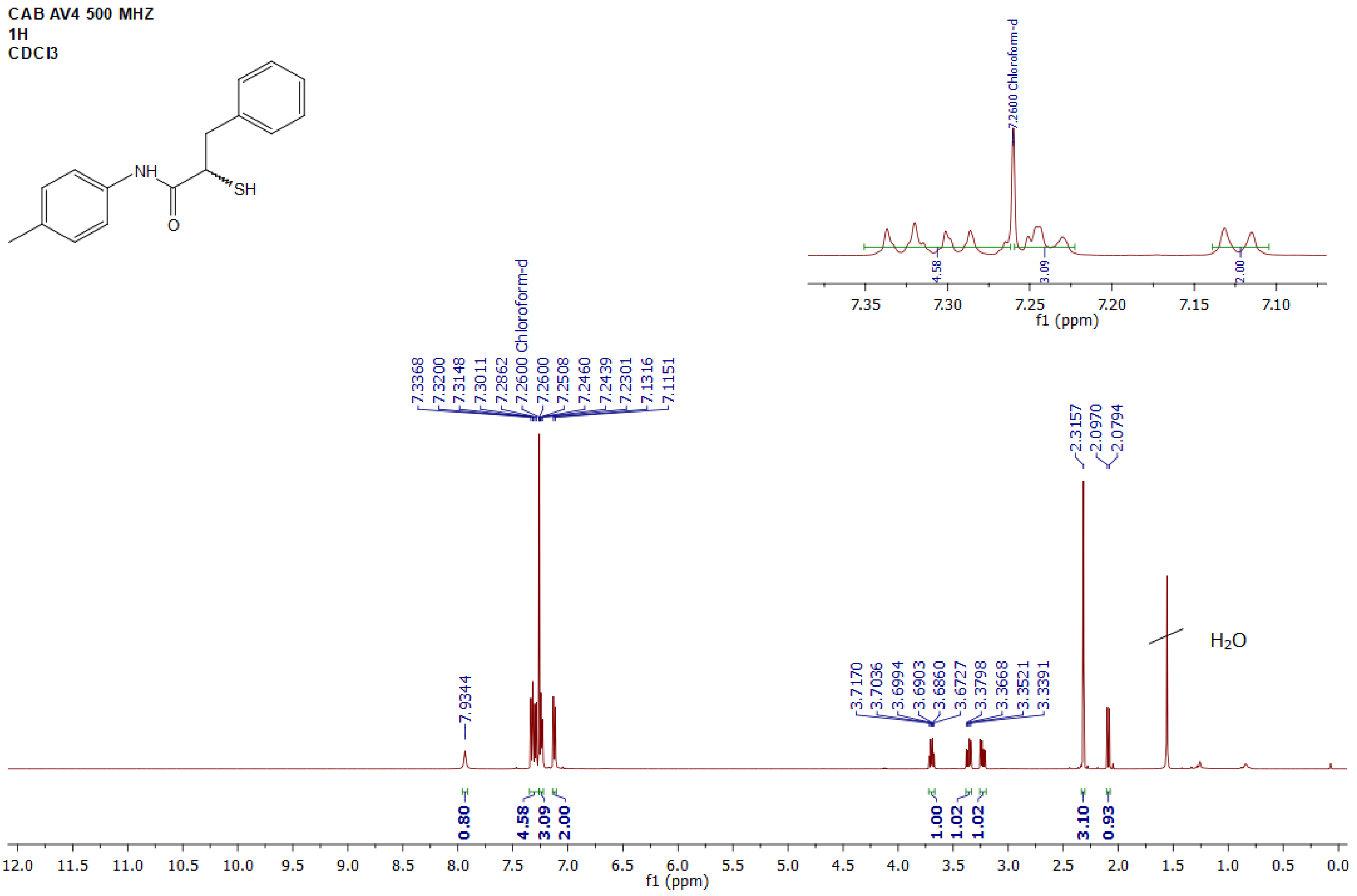

^{13}C NMR Spectra of Compoung 7g

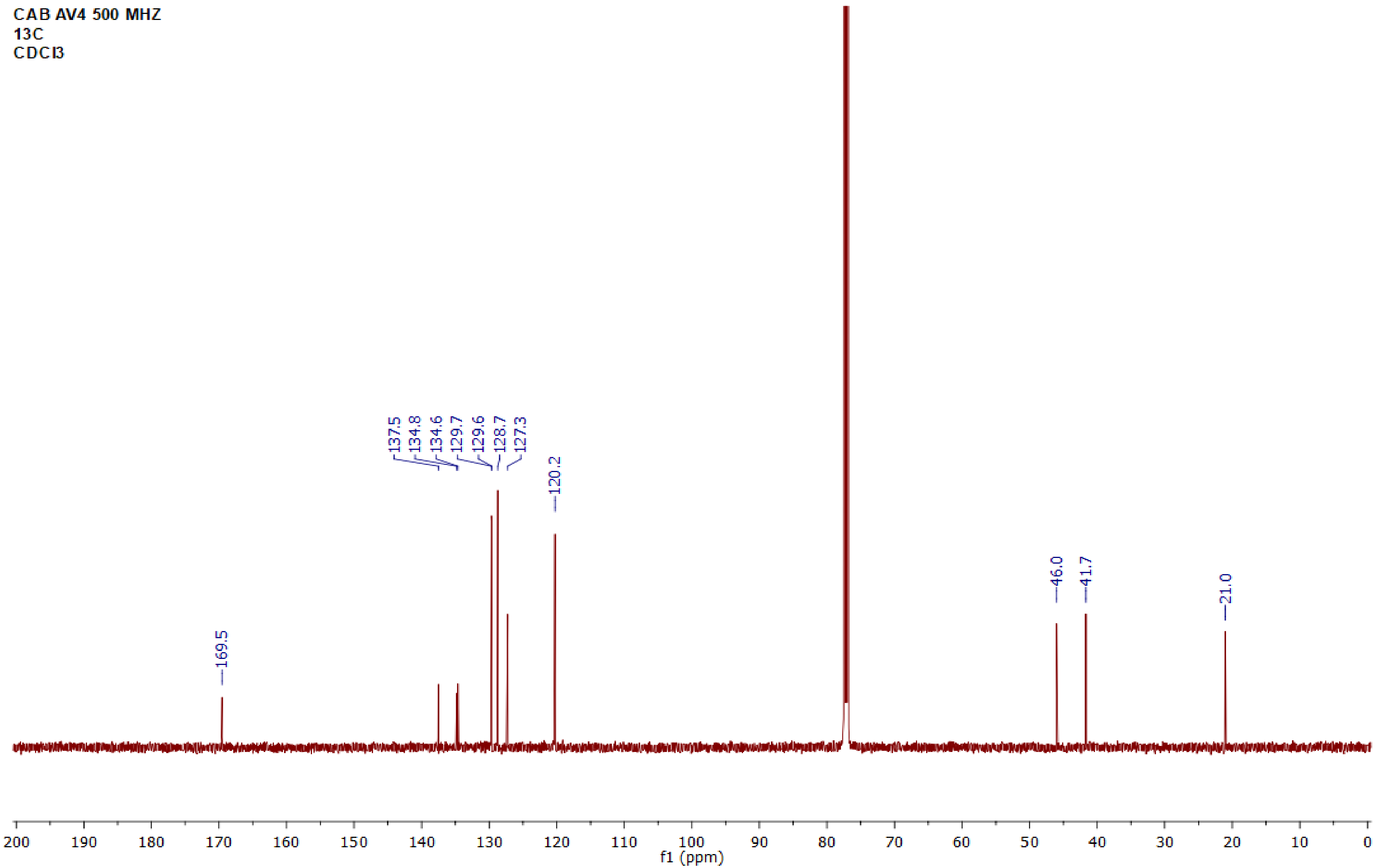

Compound	File Code
**7a**	HIPS 1726
**7b**	HIPS 5519
**7c**	HIPS 5442
**7d**	HIPS 1724
**7e**	HIPS 5390
**7f**	HIPS 5391
**7g**	HIPS 1803

Peak Analysis

Injection Details

Injection Name:	**HIPS-1726**	*Run Time (min):*	**5,10**
Vial Number:	**GC8**	*Injection Volume:*	**1,00**
Injection Type:	**Unknown**		
Calibration Level:			
Instrument Method:	**0.6ml_+ve_-ve_100-1000**		
Processing Method:	**Processing Method - New**	*Dilution Factor:*	**1,0000**
Injection Date/Time:	**12.Feb.21 16:04**	*Sample Weight:*	**1,0000**

UV

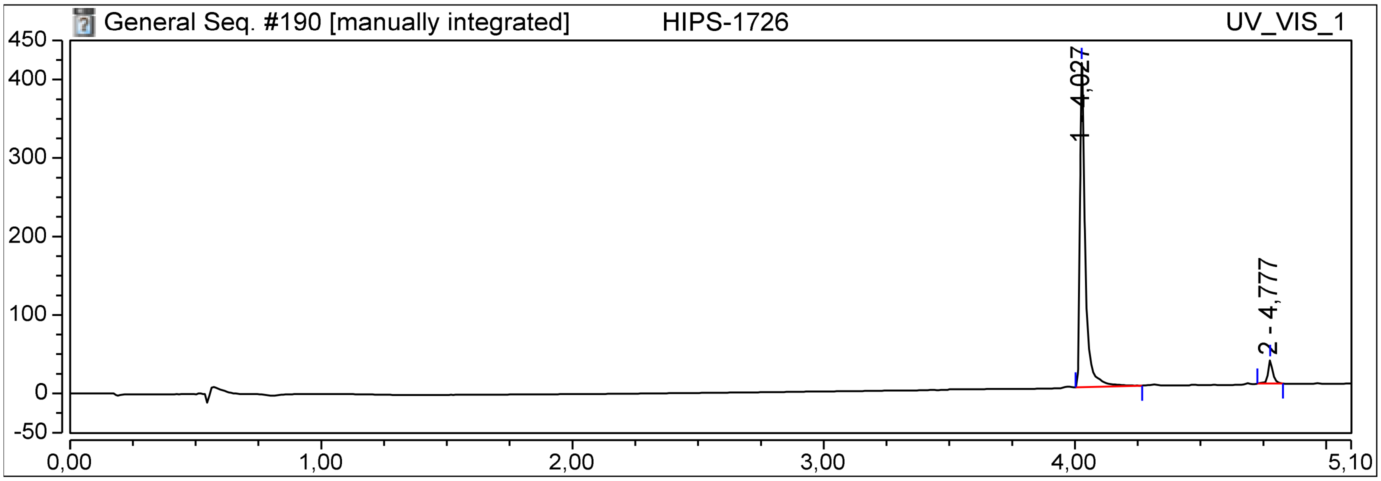

MS

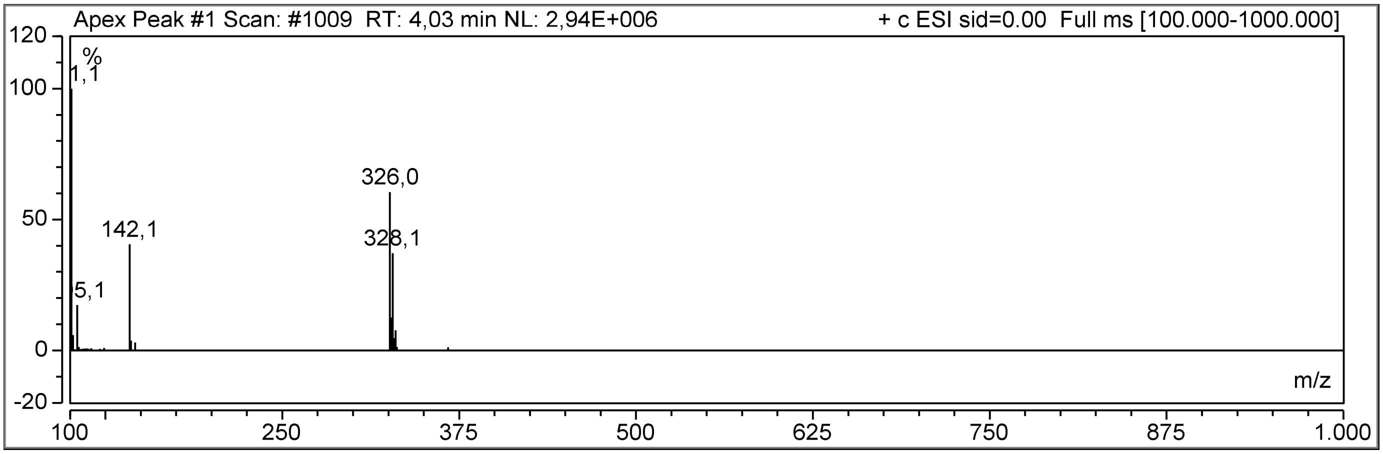

No.	Peak Name	Retention Time min	Area mAU*min	Height mAU	Relative Area %	Relative Height %
1		4,027	10,084	413,513	93,63	93,21
2		4,777	0,686	30,112	6,37	6,79

Peak Analysis

Injection Details

Injection Name:	**HIPS-5519**	*Run Time (min):*	**5,10**
Vial Number:	**GC6**	*Injection Volume:*	**2,00**
Injection Type:	**Unknown**		
Calibration Level:			
Instrument Method:	**0.6ml_+ve_-ve_100-1000**		
Processing Method:	**Processing Method - New**	*Dilution Factor:*	**1,0000**
Injection Date/Time:	**12.Feb.21 14:55**	*Sample Weight:*	**1,0000**

UV

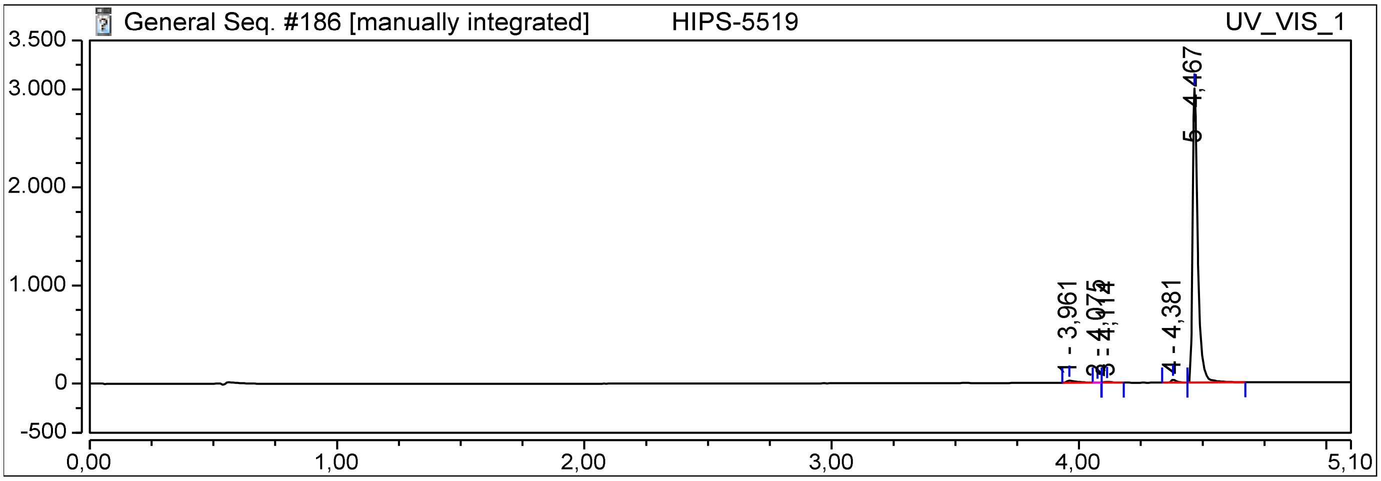

MS

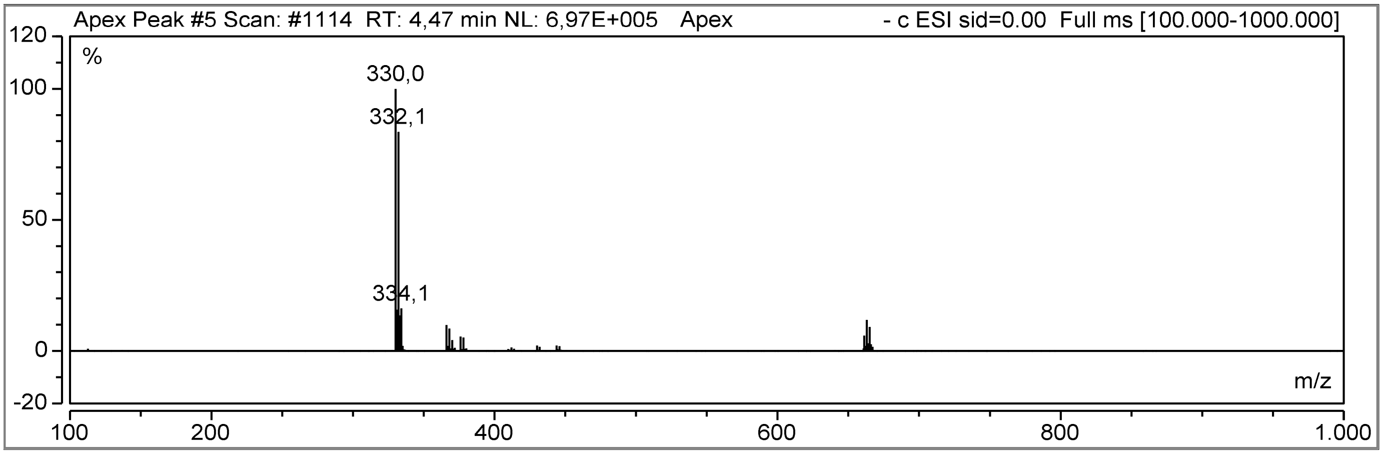

No.	Peak Name	Retention Time min	Area mAU*min	Height mAU	Relative Area %	Relative Height %
1		3,961	0,987	22,190	1,33	0,73
2		4,075	0,019	1,007	0,02	0,03
3		4,114	0,276	7,553	0,37	0,25
4		4,381	0,689	30,969	0,93	1,01
5		4,467	72,097	2997,392	97,34	97,98

Peak Analysis

Injection Details

Injection Name:	**HIPS-5442**	*Run Time (min):*	**5,10**
Vial Number:	**GC5**	*Injection Volume:*	**2,00**
Injection Type:	**Unknown**		
Calibration Level:			
Instrument Method:	**0.6ml_+ve_-ve_100-1000**		
Processing Method:	**Processing Method - New**	*Dilution Factor:*	**1,0000**
Injection Date/Time:	**12.Feb.21 14:48**	*Sample Weight:*	**1,0000**

UV

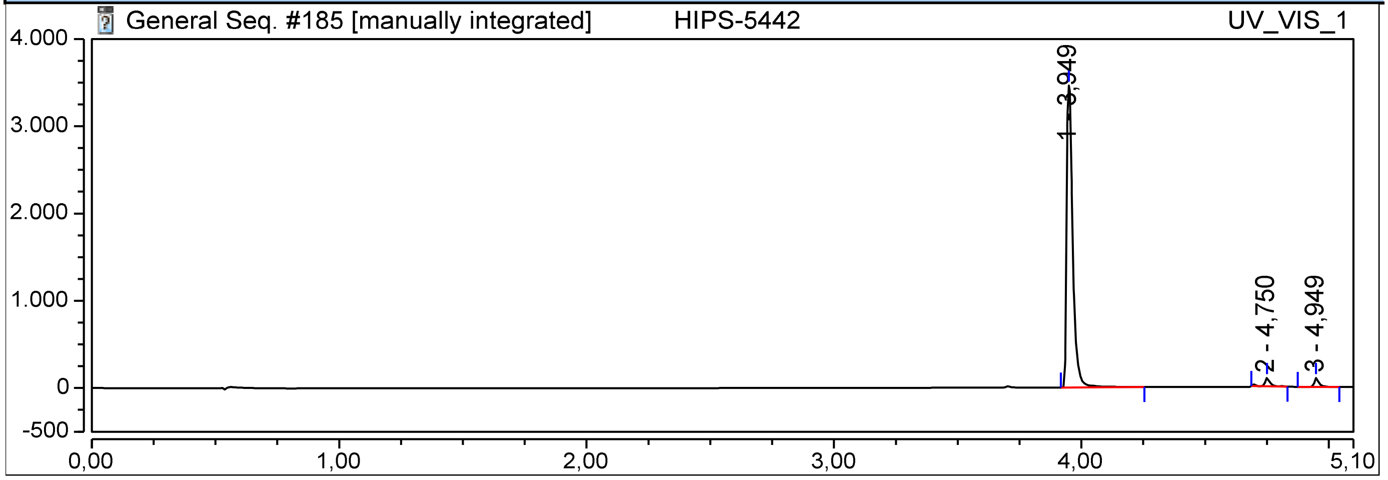

MS

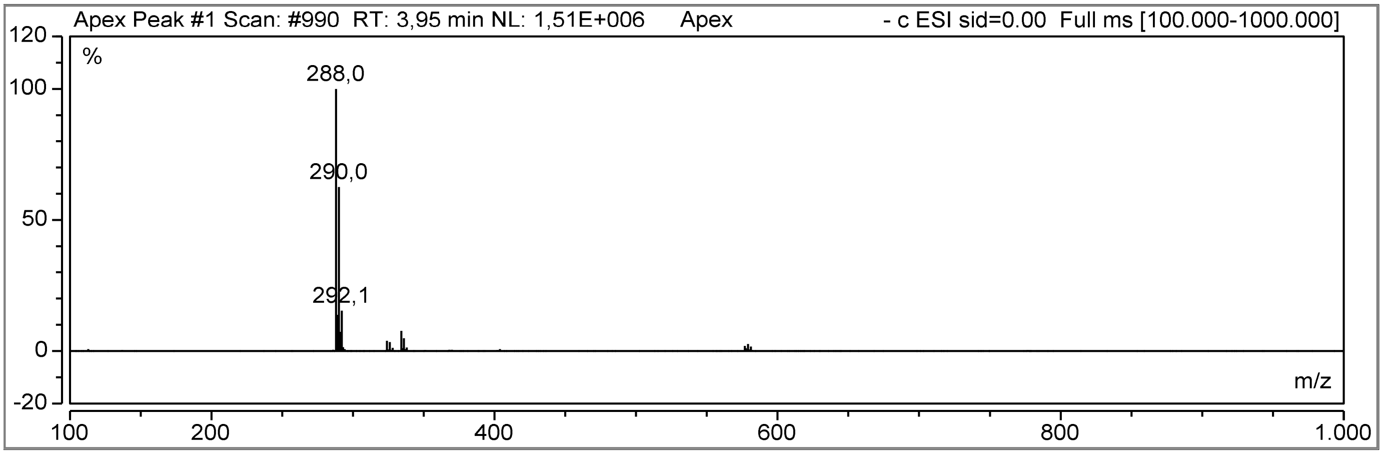

No.	Peak Name	Retention Time<br>min	Area<br>mAU*min	Height<br>mAU	Relative Area<br>%	Relative Height<br>%
1		3,949	98,377	3459,975	95,56	94,63
2		4,750	2,376	94,082	2,31	2,57
3		4,949	2,190	102,139	2,13	2,79

L:\LC-MS\...\raw\IS151Test2_181129124731   11/29/18 12:47:31

RT: 0.00 - 7.00

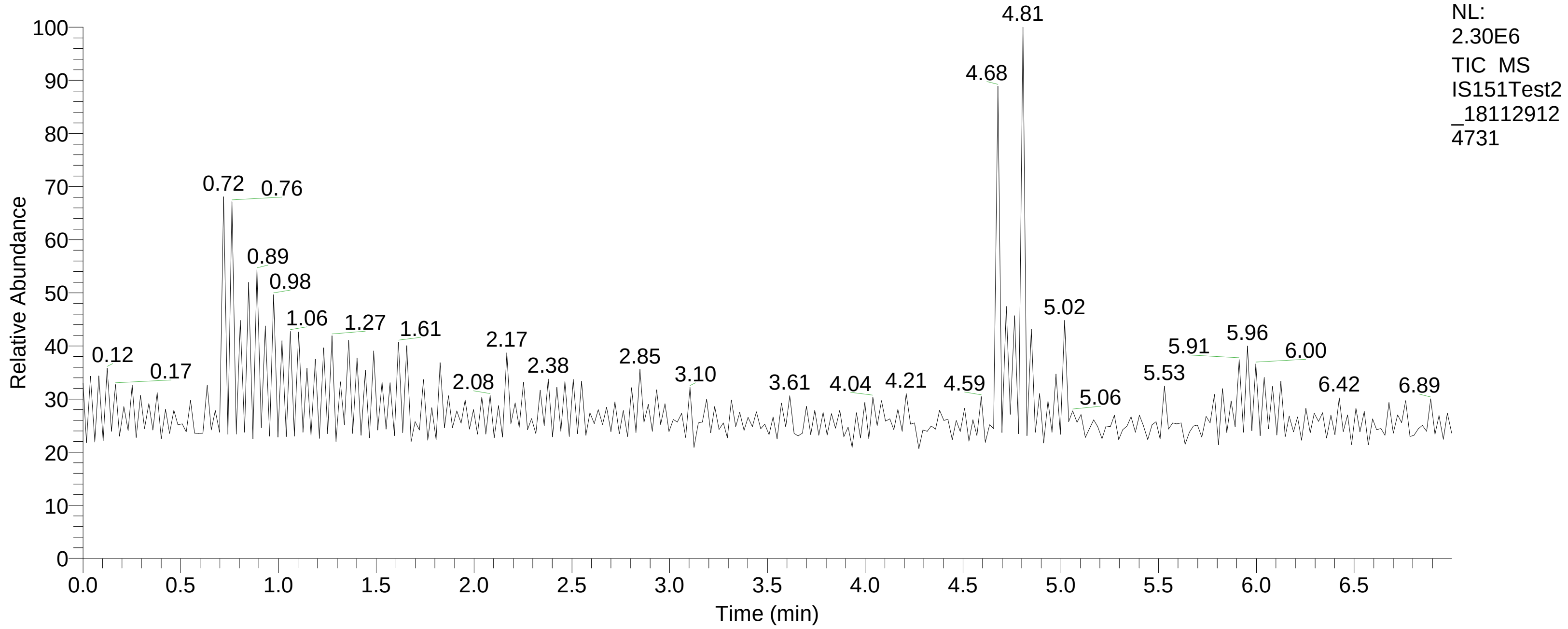

IS151Test2_181129124731 #219-246 RT: 4.64-5.21 AV: 28 NL: 7.67E3
T: {0,0} + c ESI !corona sid=55.00 det=1353.00 Full ms [100.00-1000.00]

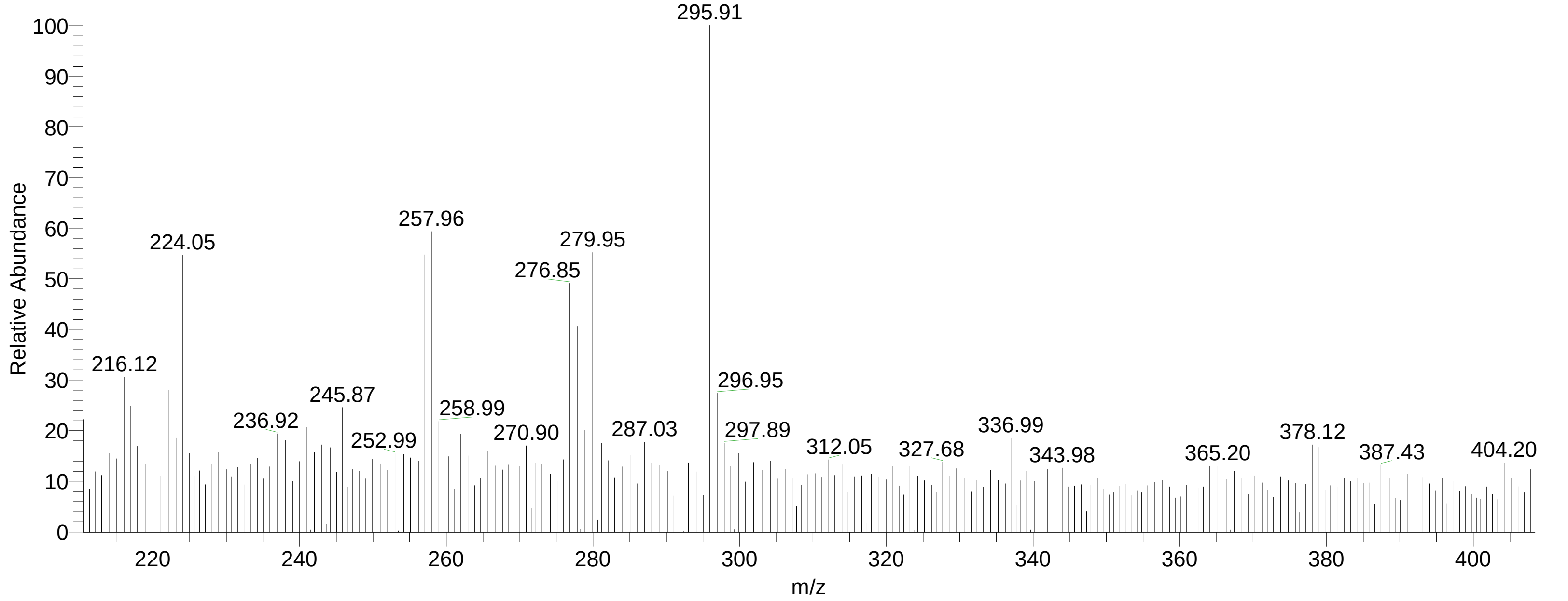

RT: 0.00 - 7.08

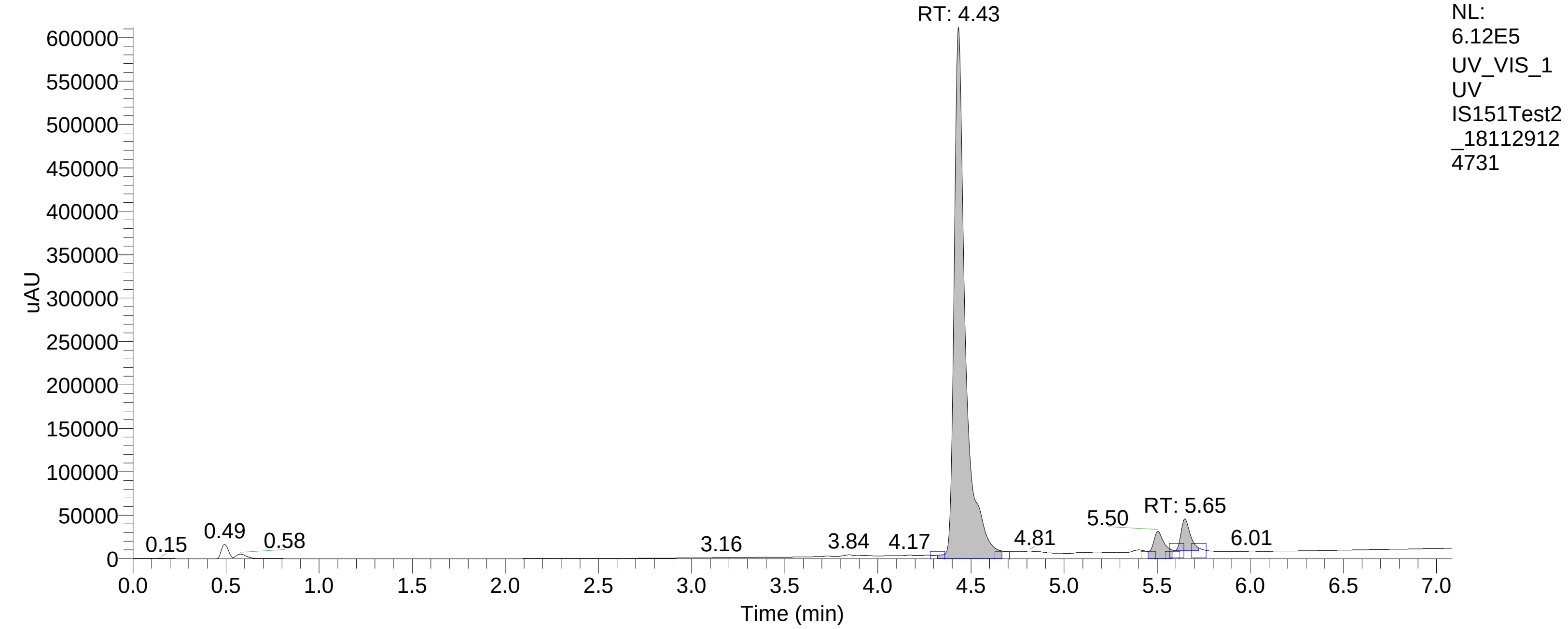

PEAK LIST

HIPS1724

RT: 0.00 - 7.08

Number of detected peaks: 3

Apex RT	Start RT	End RT	Area	%Area	Height	%Height
4.43	4.37	4.69	2549405.414	92.08	607210.801	90.74
5.50	5.45	5.58	93328.151	3.37	25088.906	3.75
5.65	5.60	5.73	126077.782	4.55	36846.953	5.51

E:\DATA\CKA\CKA-80-fr12 02/20/19 18:38:05

RT: 0.00 - 7.00

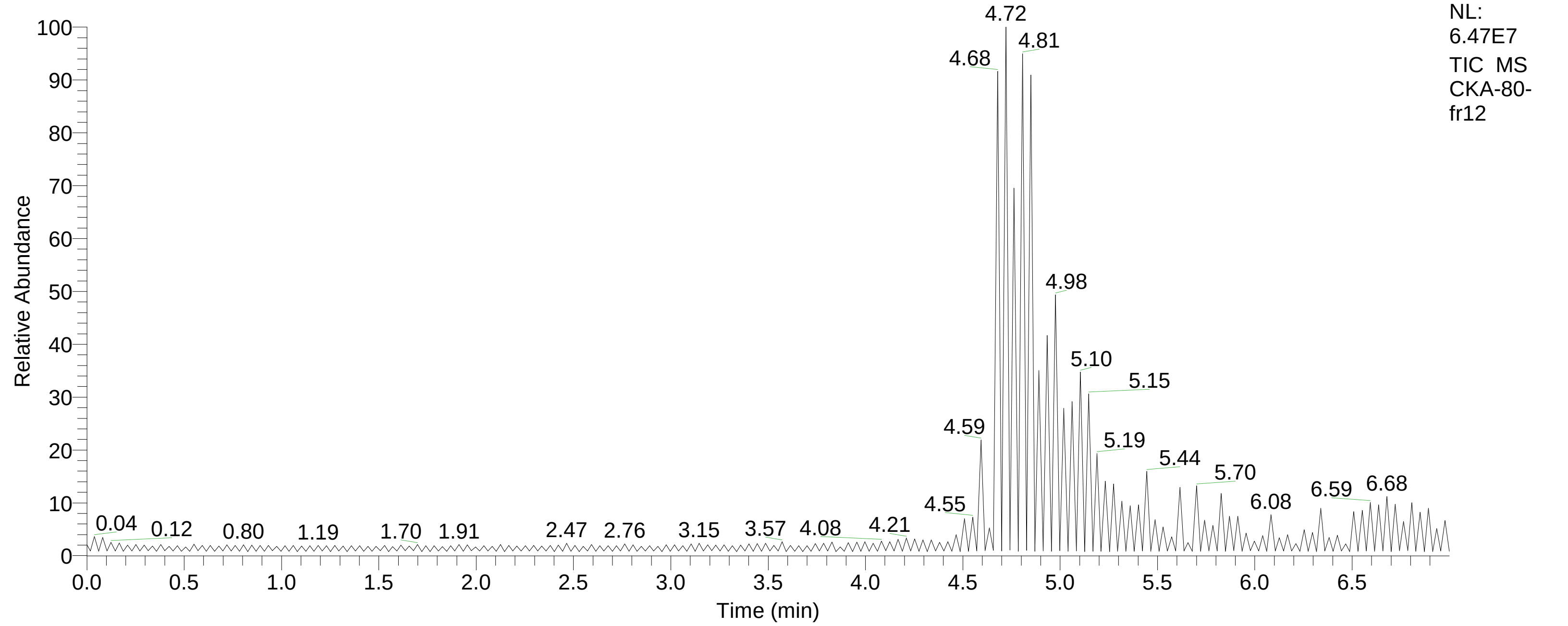

CKA-80-fr12 #196-234 RT: 4.15-4.96 AV: 39 NL: 2.69E6
T: {0,0} + c ESI !corona sid=55.00 det=1353.00 Full ms [100.00-1000.00]

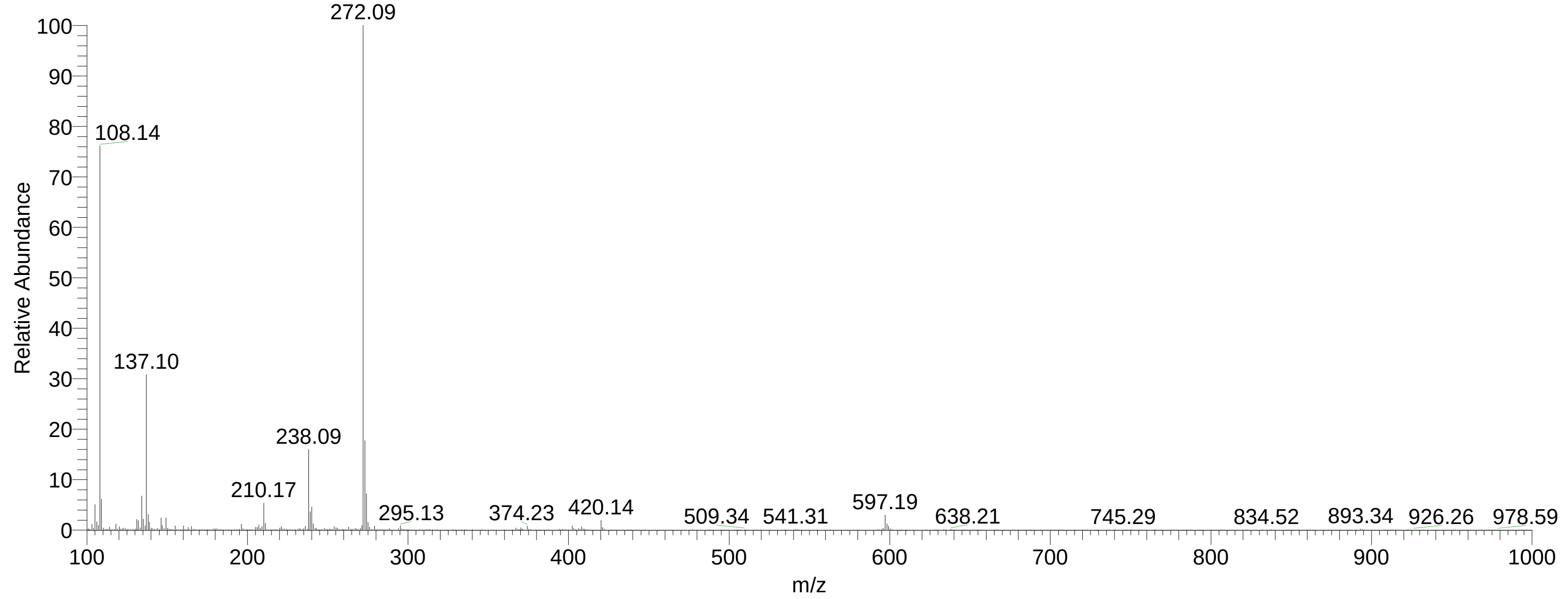

RT: 0.00 - 7.08

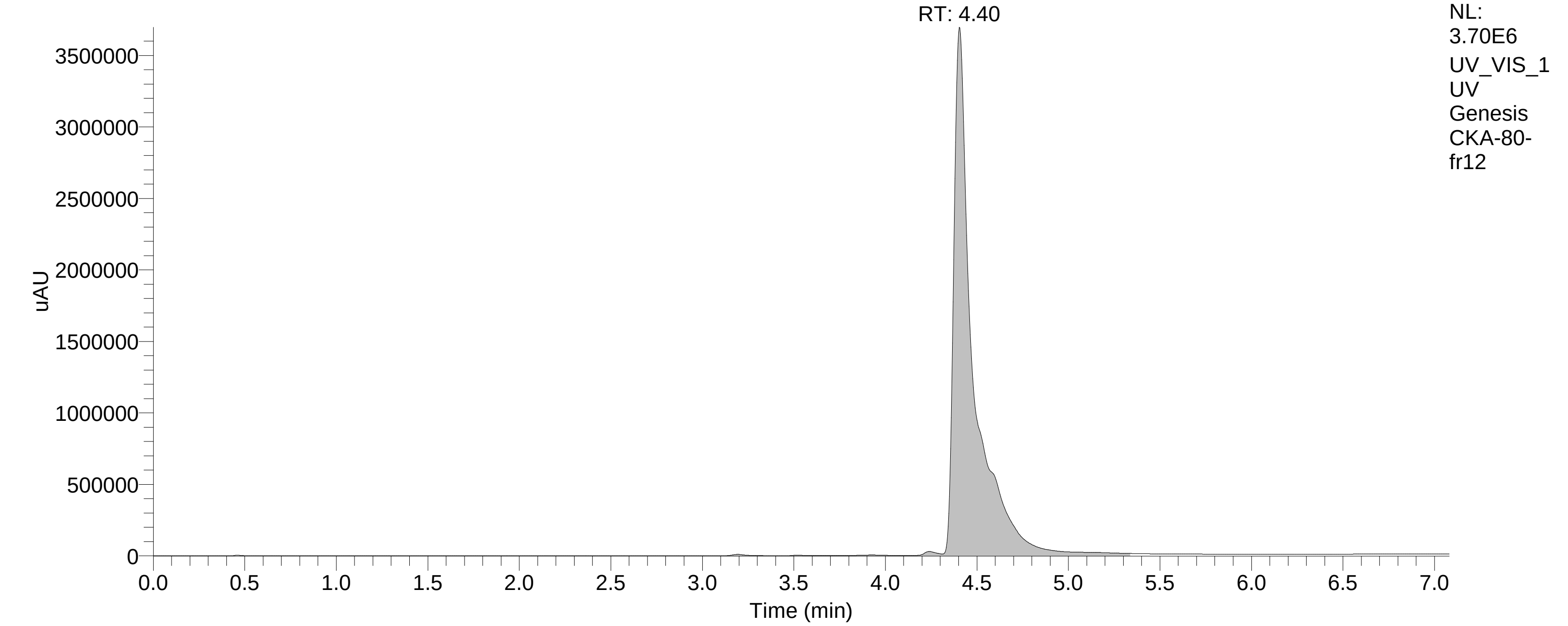

PEAK LIST

HIPS5390

RT: 0.00 - 7.08

Number of detected peaks: 1

Apex RT	Start RT	End RT	Area	%Area	Height	%Height
4.40	4.21	4.88	27000243.179	100.00	3690690.364	100.00

E:\DATA\CKA\CKA-98afterext 05/08/19 16:34:33

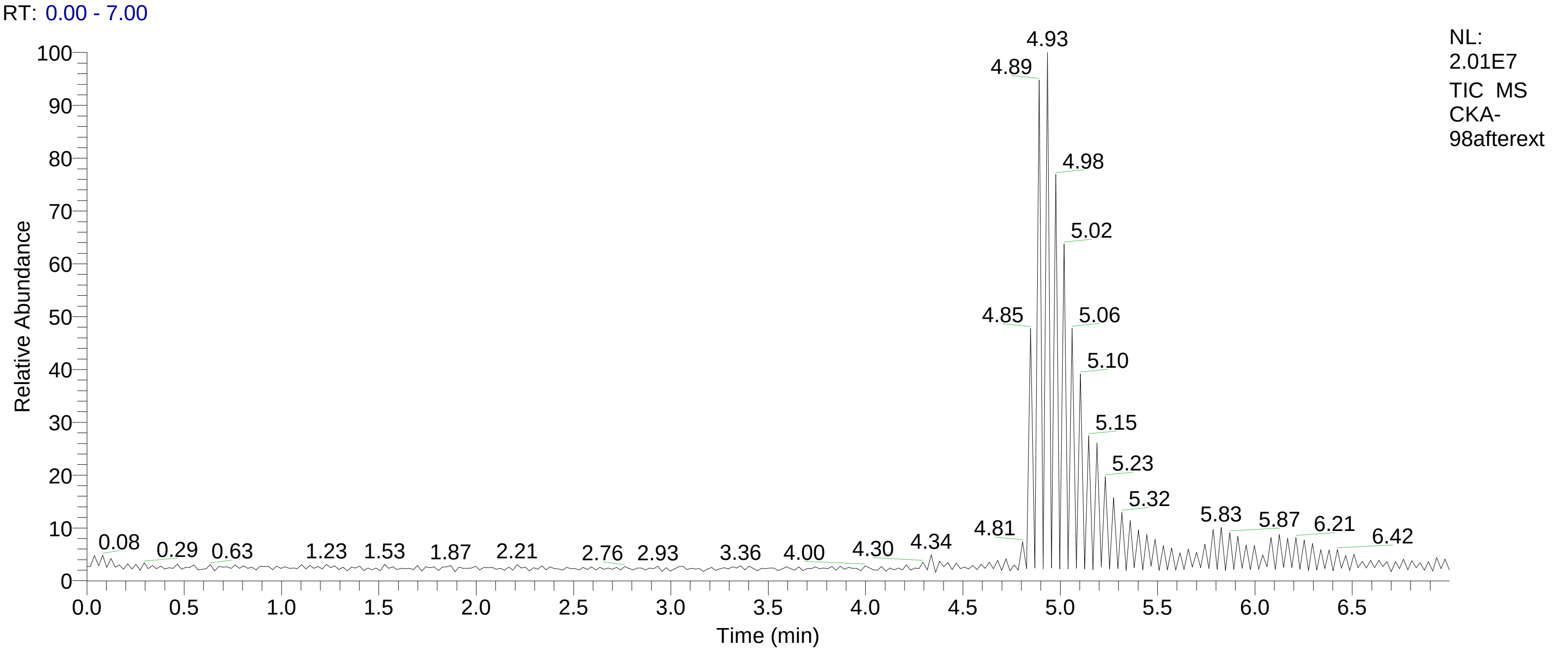

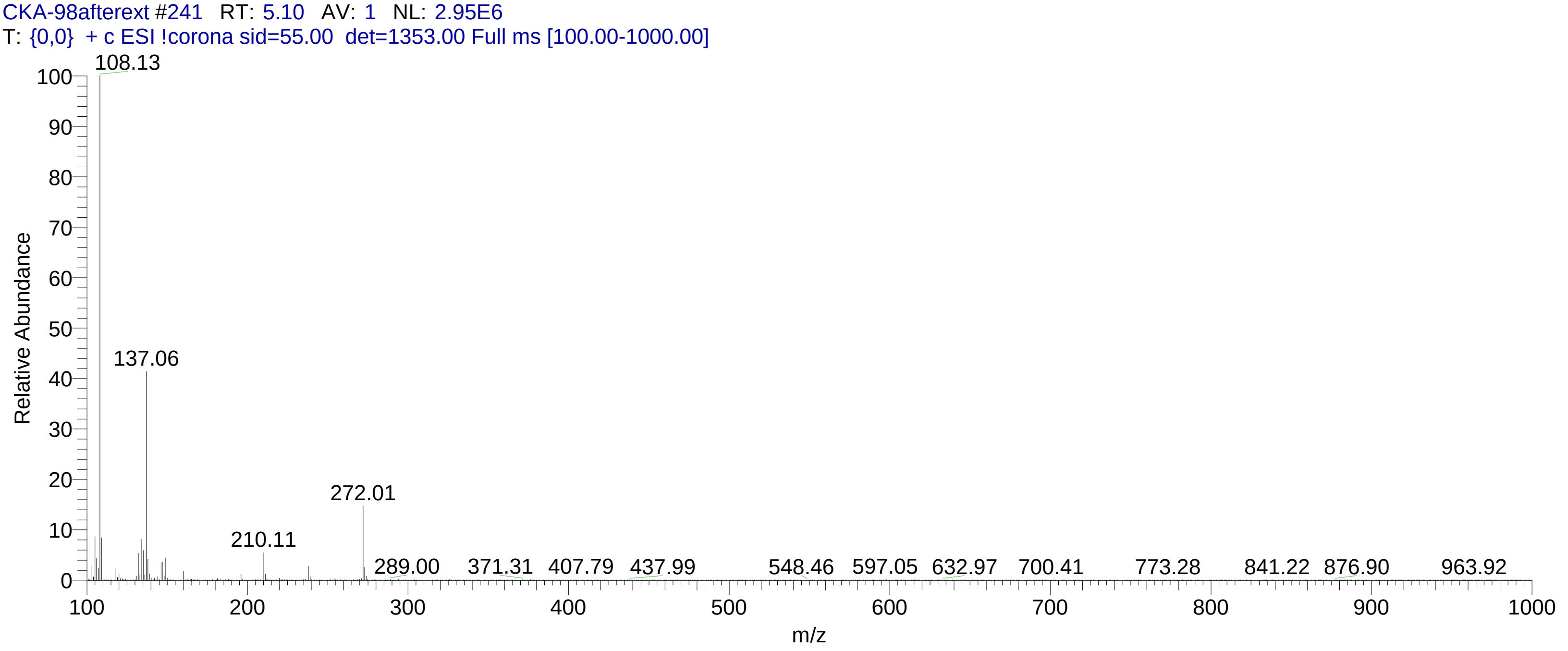

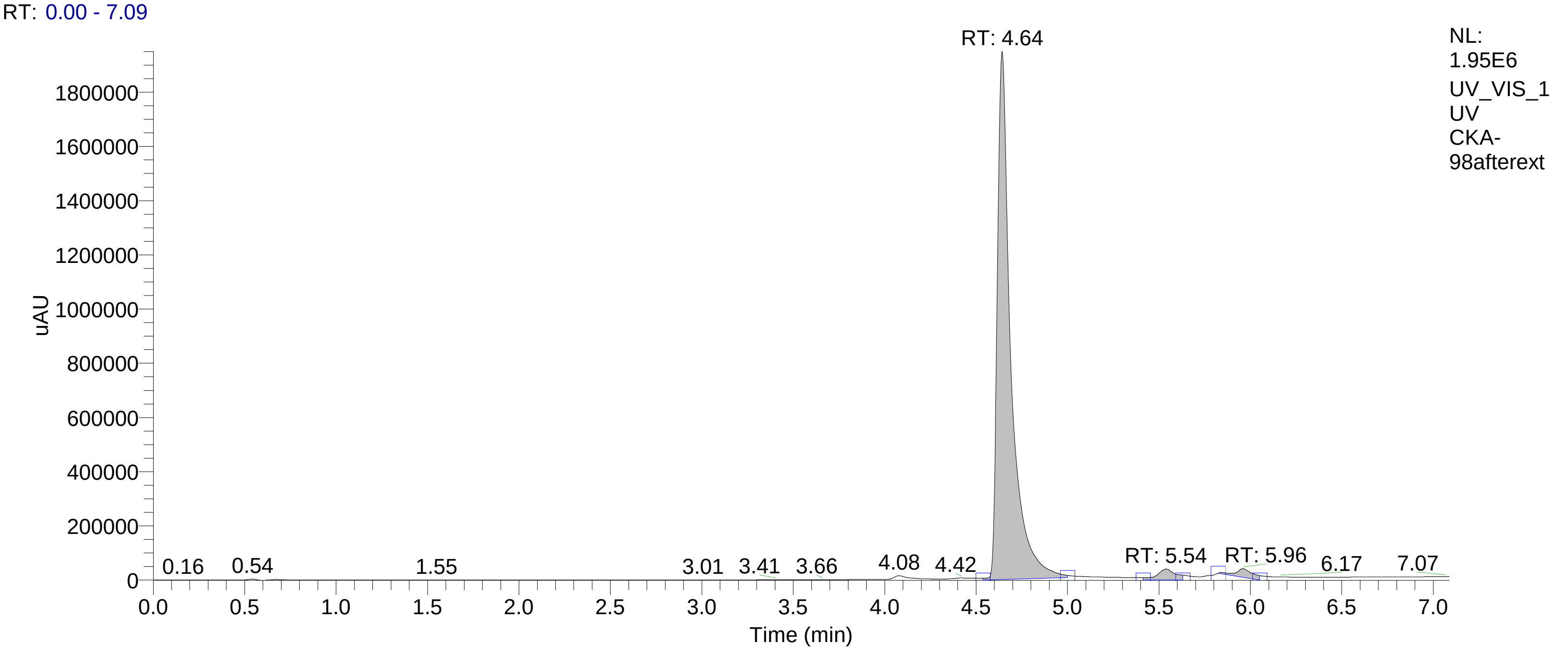

PEAK LIST

HIPS5391

RT: 0.00 - 7.09

Number of detected peaks: 3

Apex RT	Start RT	End RT	Area	%Area	Height	%Height
4.64	4.57	5.02	10056593.568	95.17	1944853.663	96.37
5.54	5.42	5.62	288094.440	2.73	40878.970	2.03
5.96	5.87	6.11	222564.479	2.11	32314.753	1.60

Peak Analysis

Injection Details

Injection Name:	**HIPS1803**	*Run Time (min):*	**5,10**
Vial Number:	**GC7**	*Injection Volume:*	**1,00**
Injection Type:	**Unknown**		
Calibration Level:			
Instrument Method:	**0.6ml_+ve_-ve_100-1000**		
Processing Method:	**Processing Method - New**	*Dilution Factor:*	**1,0000**
Injection Date/Time:	**07.Jan.21 15:54**	*Sample Weight:*	**1,0000**

UV

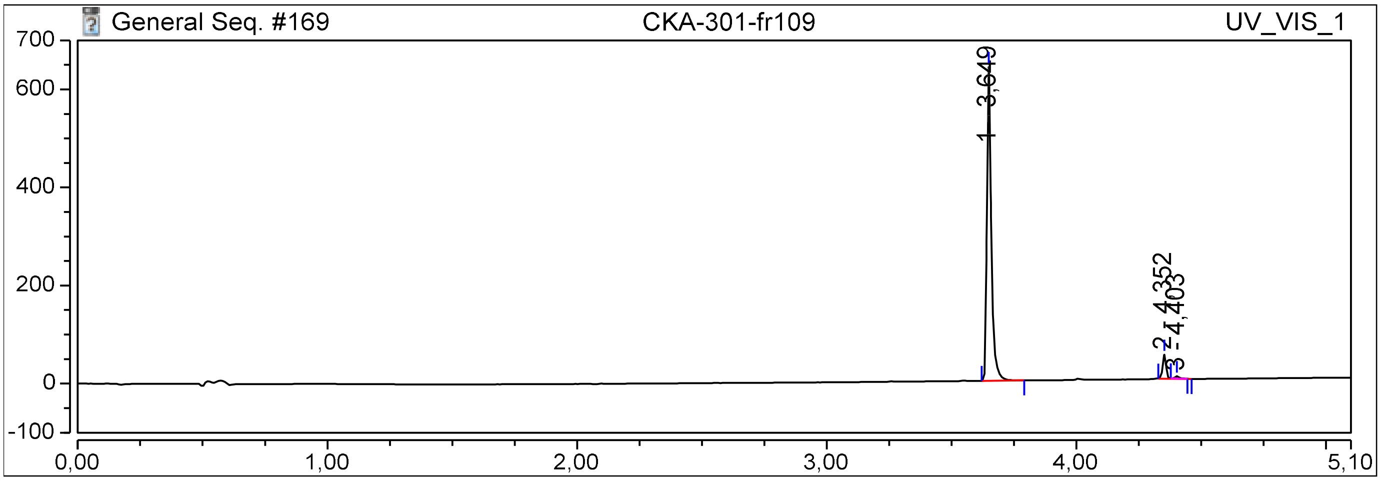

MS

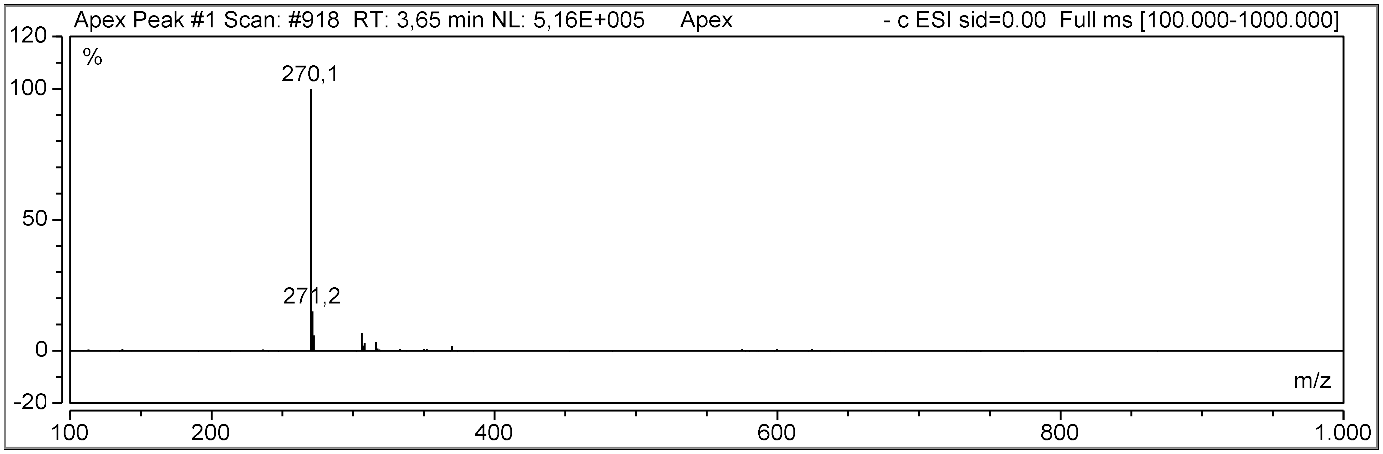

No.	Peak Name	Retention Time min	Area mAU*min	Height mAU	Relative Area %	Relative Height %
1		3,649	13,329	639,835	93,82	92,26
2		4,352	0,780	48,937	5,49	7,06
3		4,403	0,097	4,732	0,68	0,68

References

(1) Kany, A. M.; Sikandar, A.; Haupenthal, J.; Yahiaoui, S.; Maurer, C. K.; Proschak, E.; Köhnke, J.; Hartmann, R. W. Binding Mode Characterization and Early in Vivo Evaluation of Fragment-Like Thiols as Inhibitors of the Virulence Factor LasB from Pseudomonas Aeruginosa. *ACS Infect. Dis.* **2018**, *4* (6), 988–997. https://doi.org/10.1021/acsinfecdis.8b00010.

(2) Schönauer, E.; Kany, A. M.; Haupenthal, J.; Hüsecken, K.; Hoppe, I. J.; Voos, K.; Yahiaoui, S.; Elsässer, B.; Ducho, C.; Brandstetter, H.; Hartmann, R. W. Discovery of a Potent Inhibitor Class with High Selectivity toward Clostridial Collagenases. *J. Am. Chem. Soc.* **2017**, *139* (36), 12696–12703. https://doi.org/10.1021/jacs.7b06935.

(3) Kany, A. M.; Sikandar, A.; Yahiaoui, S.; Haupenthal, J.; Walter, I.; Empting, M.; Köhnke, J.; Hartmann, R. W. Tackling Pseudomonas Aeruginosa Virulence by a Hydroxamic Acid-Based LasB Inhibitor. *ACS Chem. Biol.* **2018**, *13* (9), 2449–2455. https://doi.org/10.1021/acschembio.8b00257.

(4) Haupenthal, J.; Baehr, C.; Zeuzem, S.; Piiper, A. RNAse A-like Enzymes in Serum Inhibit the Anti-Neoplastic Activity of SiRNA Targeting Polo-like Kinase 1. *Int. J. Cancer* **2007**, *121* (1), 206–210. https://doi.org/10.1002/ijc.22665.

(5) Winter, G. Xia2: An Expert System for Macromolecular Crystallography Data Reduction. *J. Appl. Crystallogr.* **2010**, *43* (1), 186–190. https://doi.org/10.1107/S0021889809045701.

(6) Kabsch, W. XDS. *Acta Crystallogr. Sect. D Biol. Crystallogr.* **2010**, *66*, 125–132. https://doi.org/10.1107/S0907444909047337.

(7) McCoy, A. J.; Grosse-Kunstleve, R. W.; Adams, P. D.; Winn, M. D.; Storoni, L. C.; Read, R. J. Phaser Crystallographic Software. *J. Appl. Crystallogr.* **2007**, *40*, 658–674. https://doi.org/10.1107/S0021889807021206.

(8) Emsley, P.; Lohkamp, B.; Scott, W. G.; Cowtan, K. Features and Development of Coot. *Acta Crystallogr. D. Biol. Crystallogr.* **2010**, *66* (Pt 4), 486–501. https://doi.org/10.1107/S0907444910007493.

(9) Adams, P. D.; Afonine, P. V; Bunkóczi, G.; Chen, V. B.; Davis, I. W.; Echols, N.; Headd, J. J.; Hung, L.-W.; Kapral, G. J.; Grosse-Kunstleve, R. W.; McCoy, A. J.; Moriarty, N. W.; Oeffner, R.; Read, R. J.; Richardson, D. C.; Richardson, J. S.; Terwilliger, T. C.; Zwart, P. H. PHENIX: A Comprehensive Python-Based System for Macromolecular Structure Solution. *Acta Crystallogr. D. Biol. Crystallogr.* **2010**, *66* (Pt 2), 213–221. https://doi.org/10.1107/S0907444909052925.

(10) Skubák, P.; Murshudov, G. N.; Pannu, N. S. Direct Incorporation of Experimental Phase Information in Model Refinement. *Acta Crystallogr. Sect. D* **2004**, *60* (12 Part 1), 2196–2201. https://doi.org/10.1107/S09074449040190.
